# Polarizable potential window at soft molecular interfaces as a quantitative descriptor for the water content in organic solvents†

**DOI:** 10.1039/d5sc00527b

**Published:** 2025-02-18

**Authors:** Siqi Jin, Lifang Yang, Sijia He, Taoxiong Fang, Xiaohang Sun, Dandan Cai, Qiong Hu, Xinjian Huang, Haiqiang Deng

**Affiliations:** a School of Chemical Engineering and Technology, Sun Yat-sen University Zhuhai 519082 China denghq9@mail.sysu.edu.cn sunxh25@mail.sysu.edu.cn; b National Engineering Research Center for Carbohydrate Synthesis, School of Chemical Engineering, Jiangxi Normal University Nanchang 330022 China; c Guangxi Key Laboratory of Agricultural Resources Chemistry and Biotechnology, College of Chemistry and Food Science, Yulin Normal University Yulin 537000 China; d Institute of Intelligent Technology, Midea Corporate Research Center Foshan 528311 China

## Abstract

The presence of water in organic solvents is a ubiquitous fact and can affect the reactivity and selectivity of chemical reactions. Traditional physical and chemical methods (IR, NMR, Karl Fischer titration, *etc.*) for quantitative measurement of water in organic solvents are not very suitable for rapid trace water analysis. Here, we demonstrate that, with hydrated Li^+^ and Cl^−^ as probes to build polarizable potential windows (PPWs) at interfaces between water and more than twenty organic solvents, we can reflect the water content in organic solvents. This method only requires a scan of a cyclic voltammogram for Li^+^ and Cl^−^ transfer (a weak-interaction electrochemical method), at a micro-scale polarized water/oil interface. A hybrid modified Born ionic solvation model was employed by us to compute the theoretical PPWs of LiCl at a series of water/oil interfaces, which match with the experimental results to some extent. Experiments and theories jointly confirm a novel and universal relationship: the PPW width correlates with the water content (in a large range) in organic solvents in a negative natural logarithm way. We postulate that when the organic solvent is different, the water fingers, *i.e.*, ions dragging a string of water molecules, will search for water molecules in the organic phase with different probabilities (or microstate numbers) after crossing the interface. This determines the macroscopic quantities, namely the standard Gibbs free energy of ion transfer and the PPW width. It is envisioned that our work paves the way for a broad spectrum of applications.

## Abstract 2

PPW width is dictated by different probabilities of ion-water finger complex encountering with water molecules in the organic phase.

## Graphical abstract

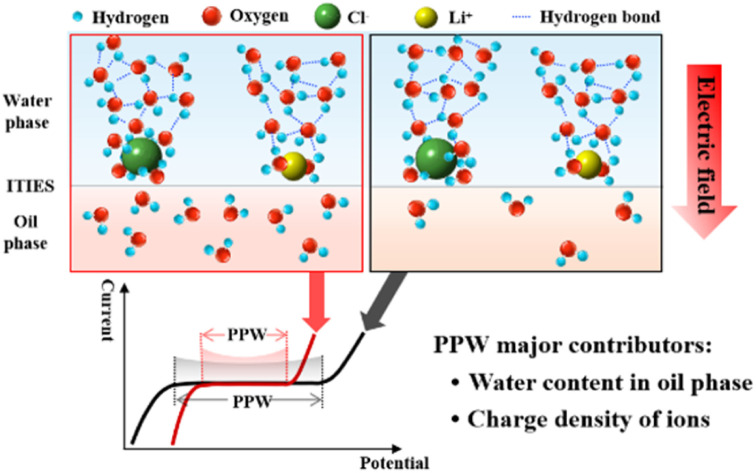

## 1. Introduction

Organic solvents are widely used in modern synthetic chemistry and many industrial production processes including liquid/liquid solvent extraction of critical elements,^1^ coating preparation, cleaning agents, and other fields. The content (even in trace level) of water, one of the most common contaminants in organic solvents, may have an important impact on chemical reactivity^2,3^ and/or product quality and performance, so accurate determination of water content is of practical significance for manufacturers to better control process parameters and ensure product consistency and quality. Traditional methods for measuring water content in organic solvents include infrared methods,^4^ nuclear magnetic resonance spectroscopy,^5^ hygrometry,^6^ distillation,^7^ electroanalysis,^8^ and the most classic Karl Fischer titration.^9–11^ Each of these methods has its own advantages and disadvantages. For example, due to the fact that exactly one molar equivalent of water *vs.* iodine is consumed in the elementary reaction in the Karl Fischer titration, it is highly quantitative, selective, and has been automated especially for the coulometric mode.^10^ However, it is a destructive technique with water as the reactant and the Karl Fischer reagent consumption is relatively high. We note that at the heart of this technique lies a simple redox chemistry (SO_2_/I_2_) and a convenient electrochemistry exploited for *in situ* generation of the Karl Fischer reagent in coulometric titration.

Due to the automation and miniaturization of electrochemical instruments, we wondered whether it would be possible to measure water content in organic solvents by using other weak-interaction electrochemistry instead of electron-transfer electrochemistry (or redox reactions)? This hypothesis is based on a well-established concept of the water finger that defined as water molecules arrange themselves into a chain or a finger due to hydrogen bonding along with the transferred high-charge-density ions across a water/oil or liquid/liquid interface following the electric field or electrochemical potential gradient. Compared with the solid/liquid interface, this interface is a defect-free, highly reproducible soft molecular interface. For the sake of rigor and convenience, we will mainly use ITIES (abbreviated for Interface between Two Immiscible Electrolyte Solutions)^12^ to refer to water/oil or liquid/liquid interface in the following text. Of course, these three terms are sometimes used interchangeably. This concept, water finger, or more precisely ion-water finger complex, is also called capillary wave fluctuation or interface roughening, firstly identified by Benjamin^13^ and later refined further by Marcus,^14^ Darvas/Jorge/Sega/Jedlovszky and their co-workers,^15^ and Morita and his co-workers.^16^

In 2021 and 2022,^17,18^ we noticed a correlation between the width of polarizable potential window (PPW, in V) at the ITIES between water and varying organic solvents and the solubility/content of water in the organic solvents, *cf.* Fig. S5† in ref. 17. Specifically, there is a negative linear relationship between the two quantities, as shown in Fig. S1 in ESI.† In our prior work, we just briefly mentioned that the saturation degree of water in organic solvents might play a key role in the formation of ionosomes.^17,18^ In the present work, we systematically calculated a number of PPW widths of ITIESs formed between (up to 23) organic solvents with different water solubility and an immiscible water solution containing LiCl. Experimentally, we used 8 water-saturated organic solvents to form micro-ITIESs^19–21^ with organic solvent-saturated water and then determined their PPW during polarization. Experiments on the evolution of PPW width by creating a water concentration gradient in the same organic solvent were also performed. Combining previous theoretical work,^15^ we proposed that the different water concentrations in organic solvents result in different probabilities of water molecules in the organic solvents meeting the ion-water finger complex from the aqueous side, thereby determining the Gibbs free energy of ion transfer and the PPW width. In this work, by classifying organic solvents, we find that the PPW width actually has a negative natural logarithm relationship with the water content (in a large range) in the organic solvent, and this relationship is universal. This work lays an experimental and theoretical foundation for measuring the water content in organic solvents by using the weak-interaction (no electron transfer and/or bond break/formation) electrochemistry.

## 2. Experimental

### 2.1 Chemicals or reagents

The following chemicals were used as received without further purification unless otherwise stated. Lithium chloride (LiCl, 99%), methanol (99.9%), 1,2-dichlorobenzene (DCB, 99.99%), 1,2-dichloroethane (DCE, 99.99%), nitrobenzene (NB, 99%), 2-nitrobenzene octyl ether (NPOE, 99%), and 5-nonanone (99%) were purchased from Aladdin. 1,6-Dichlorohexane (1,6-DCH, 99%), carbon tetrachloride (CCl_4_, 99%), tetrapropylammonium chloride (TPrACl, 97%), tetrapropylammonium tetrafluoroborate (TPrABF_4_, 98%), potassium tetrakis(pentafluorophenyl)borate (KTB, 97%), and 300–400 mesh silica gel were purchased from Macklin. α,α,α-Trifluorotoluene (TFT, 99.5%) was sourced from Alfa Aesar. Hydrogen peroxide solution (H_2_O_2_, 3% w/w), tetraethylammonium chloride (TEACl, 98%), and tetradodecylammonium bromide (TDDABr, 99.0%) were bought from Sigma-Aldrich. Sulfuric acid (H_2_SO_4_, 98%) and hydrochloric acid (HCl, 36–38%) were provided from Xilong Scientific (Shantou, China). Bis(triphenylphosphoranylidene)ammonium chloride (BACl) was ordered from Fluka. Lithium tetrakis(pentafluorophenyl)borate diethyl etherate (LiTB) was purchased from Boulder Scientific, USA. The mainly used lipophilic/hydrophobic supporting electrolyte of bis(triphenylphosphoranylidene)ammonium tetrakis(pentafluorophenyl)borate (BATB) was synthesized and purified according to the method reported elsewhere.^3^ Another one, *i.e.*, tetradodecylammonium tetrakis(pentafluorophenyl)borate (TDDATB), was used as a supporting electrolyte in CCl_4_ (also in TFT, *vide infra*). It was prepared by a metathesis reaction in a 1 : 1 molar ratio between TDDABr and KTB, followed by a purification (silica gel chromatography combined with rotary evaporation) process.^22^ All aqueous solutions were prepared using Millipore-Q ultra-pure water (≥18.2 MΩ cm). The PHS-3 C pH meter (Shanghai Leici Company, China) was employed to measure the pH values of the aqueous solutions.

Since the purpose of this work is to study the relationship between the PPW width of micro-ITIES and the water contents in the organic solvents, we prepared organic solvents with different water contents (see “Regulation of Water Content in Organic Solvents”, ESI,† for details). The results of the organic solvents saturated with water are shown in Fig. 3 and 4 and the relevant discussion in the main text (see below); the results of different water contents in the same organic solvent are shown in Fig. 5 and the relevant discussion in the main text (see below).

### 2.2 Micropipettes fabrication and characterization

**Scheme 1:**
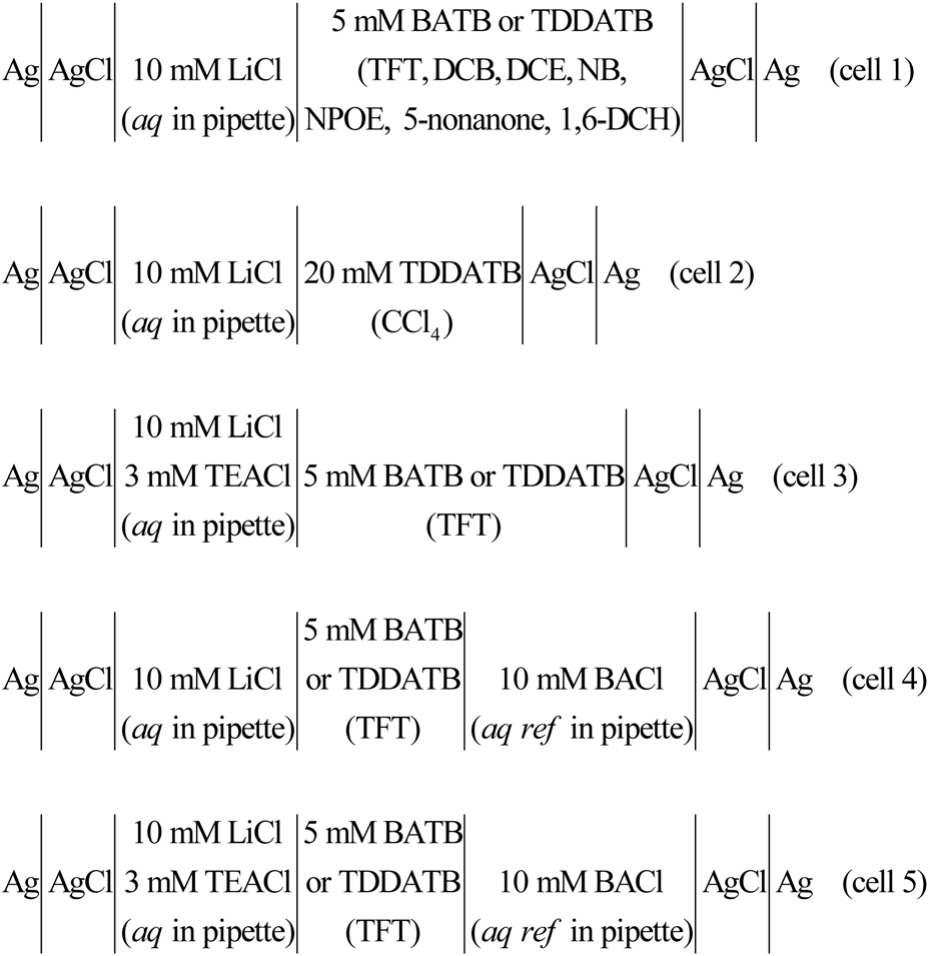
The electrochemical cell composition. The *aq ref* denotes the aqueous reference phase for the organic phase.

We used a PC-100 hot wire puller (Narishige Instrument, Japan) to pull borosilicate glass capillaries (outer and inner diameters, abbreviated as o.d. and i.d. throughout the text: 1.0/0.58 mm, length 10 cm, with filament; Sutter Instrument, USA) into short-shank patch-type glass micropipettes. The i.d. of the micropipettes used for supporting the ITIES in this work ranges from 1 to 5 μm, with 1–1.5 μm (*e.g.*, a 1.2 μm micropipette shown in Fig. S2A, ESI†) being the most common. The one-stage pulling parameters were applied to fabricate such a micropipette: heater (no. 1) value: 47–50, number of load-bearing weights: 2–4. We also used a laser-based P-2000 puller (Sutter Instrument Co.) to pull quartz glass capillaries (o.d./i.d.: 1.0/0.5 mm, length 10 cm, without filament; Sutter Instrument, USA) into short-shank patch-type larger micropipettes with typical diameters ≥10 μm (10–20 μm; *e.g.*, a 11.5 μm micropipette shown in Fig. S2B, ESI†). This larger micropipette was filled with an aqueous reference solution (*i.e.*, “*aq ref* in pipette”, see electrochemical cells 4 and 5 in Scheme 1) and placed within the oil phase for a better reference potential control at the oil/*aq ref* interface. We wrote a multi-line program to pull such a quartz glass micropipette, with the specific parameters exemplified as follows: Line 1: HEAT = 525, FILAMENT = 4, VELOCITY = 25, DELAY = 225, PULL = 0; Line 2: HEAT = 525, FILAMENT = 4, VELOCITY = 24, DELAY = 225, PULL = 0; Line 3: HEAT = 525, FILAMENT = 4, VELOCITY = 22, DELAY = 225, PULL = 0; Line 4: HEAT = 525, FILAMENT = 4, VELOCITY = 22, DELAY = 225, PULL = 0; Line 5: HEAT = 525, FILAMENT = 4, VELOCITY = 22, DELAY = 225, PULL = 0. Before pulling, very carefully clean the inner walls of the borosilicate/quartz glass capillaries with piranha solution. Then rinse with plenty of water and put them in the oven to dry. Note: when the water pH is close to neutral (slightly acidic), the capillaries are clean. Usually, the size, tip morphology, and taper angle of micropipettes, were scrutinized by a metallographic optical microscope (model: IE31, Mshot Co., Guangzhou, China). We also imaged orifice of a typical micropipette by a field-emission scanning electron microscope (SEM, Hitachi SU 8010, Japan, 3 or 5 kV accelerating voltage). To minimize the charging effect, a gold nanofilm was sputtered on the shank region of a micropipette by a gold sputtering device prior to SEM imaging.

### 2.3 Electrochemical measurements

All electrochemical measurements were performed in a single-compartment electrolytic cell with a two-electrode system (Scheme 1 and Fig. S3 in the ESI†). Information on electrochemical measurement instrument (*i.e.*, potentiostat) and Faraday cage for shielding (*e.g.*, electrical) noise can be found in our previous work.^18,23,24^ The electrodes connected to the potentiostat were all Ag/AgCl wires, and their dimensions and preparation methods were also seen in our previous work. The micropipette was filled with an aqueous electrolyte solution (by a 10 μL syringe, World Precision Instruments) and a Ag/AgCl wire was inserted as the working electrode (WE), while the outside of the micropipette was filled with an oil electrolyte solution and another Ag/AgCl wire was put inside and functioned as the pseudo counter/reference electrode (CE/RE). This experimental configuration is specified in cells 1–3 in Scheme 1 and illustrated in Fig. S3A in the ESI.† There is an aqueous reference solution for the oil phase, as detailed in cells 4 and 5 of Scheme 1 (and also illustrated in Fig. S3B in the ESI†), in order to confirm the reliability of cells 1–3 (see Results and discussion for more details). We assume that the interface is formed at the pipette orifice. According to the IUPAC, currents that transfer positive charges from the aqueous phase into the oil phase or negative charges in the opposite direction are recorded as positive.

## 3. Results and discussion

### 3.1 Theory

The hydration of high-charge-density ions in organic solvents is a known demonstrable fact.^2,17^ In aqueous phase for a hydrated ion *i*, the standard transfer (to organic phase) potential of *i*, 
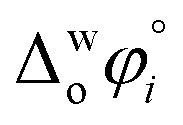
, equals to1
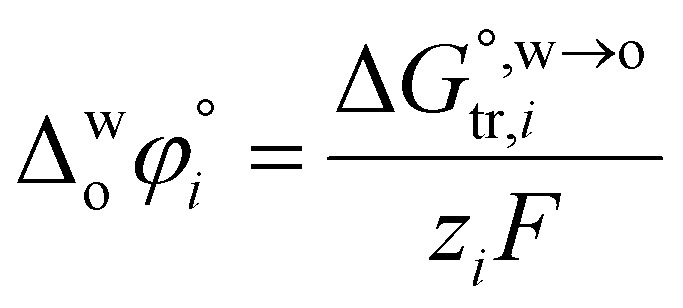
in which 
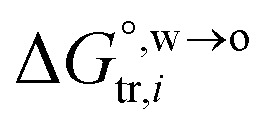
 is the standard Gibbs energy of *i* transfer from aqueous to organic phase. At room temperature and 1 atm pressure, 
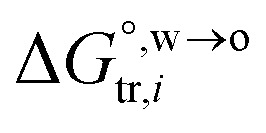
 can be further expressed as:2
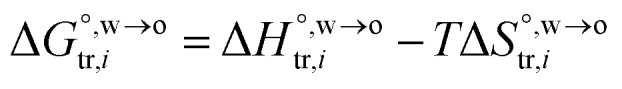
where 
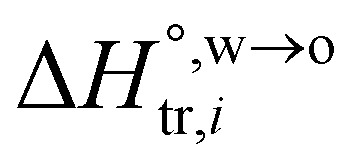
 and 
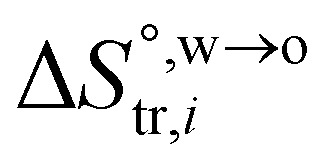
 are standard enthalpy change and entropy change of *i* transfer from aqueous to organic phase, respectively. According to the theoretical work of Darvas/Jorge/Sega/Jedlovszky and their co-workers,^15^ the interfacial water concentration from the oil side dictates the Gibbs free energy of hydrated ion (*e.g.*, Li^+^ and Cl^−^) transfer. We selected Cl^−^ and Li^+^ ions as limiting ions for controlling the width of PPW, and the Gibbs free energies of their transfer are positive. There are some reasons for choosing them,^25^ such as: (1) Cl^−^ and Li^+^ ions are stable species when hydrated in organic solvents; (2) the charge density is large enough to maintain the relative stability of the hydration layer; (3) Cl^−^ ions are much less toxic than fluoride ions. It is known that the dissolution of an ionic compound like LiCl in a solvent includes several steps: (1) breakup of solute–solute attractions (endothermic); (2) breakup of solvent–solvent attractions (endothermic); (3) formation of solvent–solute attractions (exothermic). Considering water has a dielectric constant different from (more precisely, say, bigger than) organic solvents, the enthalpy change, 
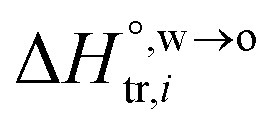
, for ion transfer from water to an immiscible organic solvent, is normally positive. For the same reason, however, the entropy change of ion solvation, 
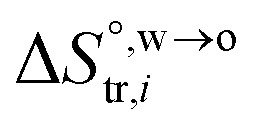
, is negative, and as the polarity of the organic solvent decreases, the absolute value of entropy reduction increases (see the work of Abraham *et al.*, Table 4 (ref. 26)). Looking back at eqn (2), we can conclude that the Gibbs free energy for ion transfer, 
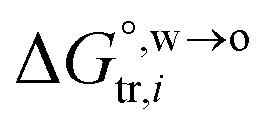
, from the aqueous phase into the less polar, immiscible organic solvent is greater. It means that the standard transfer potential of the ion is more positive (for cations) or more negative (for anions). This potential mirrors the interfacial water concentration at the oil side of an ITIES, which is proportional to the concentration of water molecules in the bulk of the organic medium. Experimentally, the PPW width is equal to the difference between the onset transfer potentials of Li^+^ and Cl^−^ ions at the ITIES, plus a constant value of 0.15 V (refer to Fig. S4 and its relevant text in the ESI†). Note that it is not necessary to correct the interface potential (difference) to the Galvani scale, since not all organic solvents forming ITIESs with water that can be corrected to the Galvani scale, and the difference between the onset transfer potentials of Li^+^ and Cl^−^ ions does not require potential correction.

**Fig. 1:**
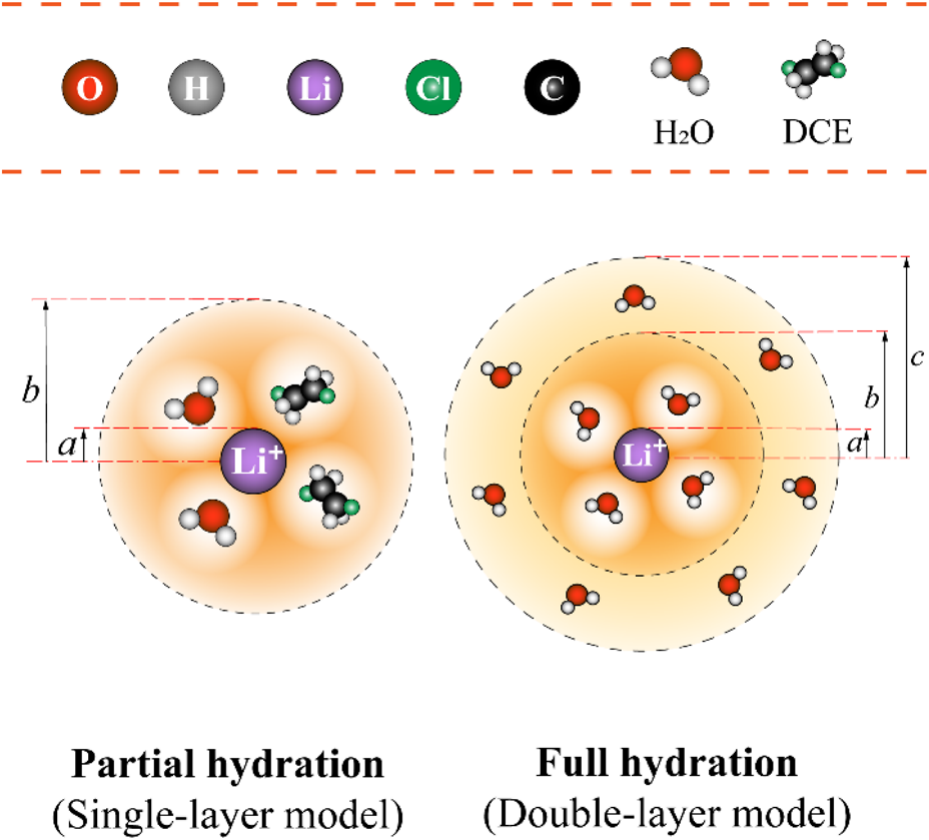
The modified single-layer (lower left) or double-layer (lower right) Born model (not to scale) of an ion solvated by water (and/or organic solvent) molecules that are then immersed within the bulk of organic solvents featured with either low or high water content, respectively. Regarding the latter (*e.g.*, aniline), the ion has a radius of *a* and is surrounded by a first layer of water molecules having a thickness of *b* − *a* and a second layer of water molecules having a thickness of *c* − *b*. Note: for organic solvents with low water content (here, take the DCE as an example), a single water (and/or organic solvent) solvation layer (thickness *b* − *a*) model is used.

Let's proceed to the modelling of ionic solvation of a hydrated ion within an organic medium. According to the low and high water content of the organic solvents (denoted as “dry” and “wet” solvents, respectively), Li^+^ and Cl^−^ are partially or fully hydrated. Correspondingly, the one-layer and two-layer hydration layer models in Fig. 1, respectively, are used to calculate the Gibbs solvation (and transfer) energy of these two ions. In fact, this is the modified version of Born ionic solvation model,^27^ which was proposed by Abraham *et al.*^26,28,29^ and Marcus,^30,31^ respectively. Briefly, a spherical ion of radius *a* is surrounded by a layer of oriented solvent molecules with thickness *b*–*a* and local dielectric constant *ε*_1_. In the case of hydrated Li^+^ and Cl^−^, the oriented solvent molecule is water. For some “wet” solvents like aniline and *n*-octanol (*n*-ocT) (*cf.* Table S1, in the ESI†), the existence of a second (but less structured) hydration layer (with thickness *c*–*b* and local dielectric constant *ε*_2_, see Fig. 1) is quite probable. Then, the concentric spheres shown in Fig. 1 are immersed as a whole in a bulk organic solvent having a dielectric constant *ε*_0_. In the present work, the organic solvents are categorized into three main groups: halogenated hydrocarbons, aromatic hydrocarbons, and fatty ketones/alcohols. This classification takes into account the differing structures of solvents and potential effects that hydrogen-bonding solvents might induce. Detailed information regarding the physical and chemical properties of these solvents at 293 K can be found in Table S1 in the ESI.†

Now, the standard Gibbs free energy of ion transfer from the aqueous phase to the oil phase can be represented by formula (3),3
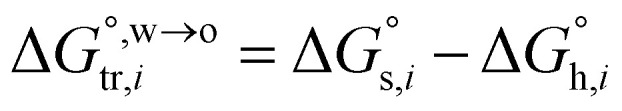
where 
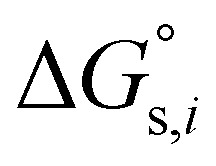
 and 
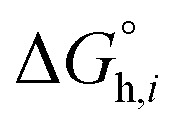
 denotes the Gibbs solvation energies of the ion *i* in organic solvents and water, respectively. The latter, *i.e.*, the hydration free energy, is typically known and compiled in Table S2 in the ESI† (see the 5th column from the left). Thus, we need calculate the Gibbs solvation free energy of the ion in the solvents other than water. 
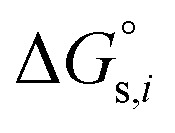
 can be divided into electrostatic term, 
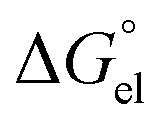
, and neutral term, 
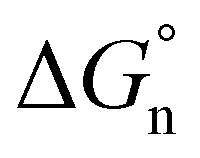
, with the formula shown below:4
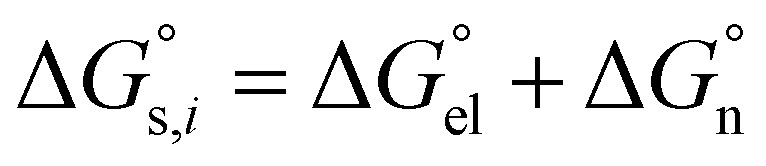


For solvation of Li^+^ and Cl^−^ in relatively “dry” organic solvents, the first solvation layer (see *b*–*a*, Fig. 1) may not be composed exclusively of water molecules. In this case, the single-layer model for calculating the electrostatic term (in kJ mol^−1^) is formulated in eqn (5).5
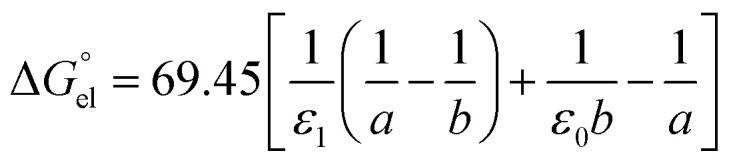
In eqn (5), *ε*_1_, *ε*_0_, *a* and *b* have been defined previously; specifically, *ε*_1_ = *n*_D_^2^ (listed in the 5th column from the left, Table S1, ESI†), where *n*_D_ is the refractive index of organic solvents; *ε*_0_ is the dielectric constant of solvents that is listed in the 4th column in Table S1, ESI;† and *a* refers to the radii of the naked ions, being 0.078 nm and 0.181 nm for Li^+^ and Cl^−^ (*cf.* Table S2, ESI†), respectively. 69.45 = *e*^2^*N*_A_/8π*ε*, in unit of kJ nm mol^−1^, in which *e* is the elementary charge (1.602 × 10^−19^ C), *N*_A_ is the Avogadro constant (6.022 × 10^23^ mol^−1^), and *ε* is the dielectric constant or permittivity of the free space/vacuum (8.854 × 10^−12^ F m^−1^). Considering the possible composite nature of the first solvation shell of Li^+^ or Cl^−^ ions in the relatively “dry” organic solvents, the dielectric constant *ε*_1_ in eqn (5) is replaced by the weighted average value 
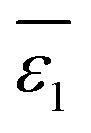
, as depicted in eqn (6).6
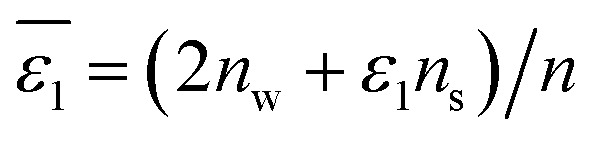
In eqn (6), *n*_w_ + *n*_s_ = *n*, in which *n* is the total number of solvent molecules in the first solvation shell, and *n*_w_ and *n*_s_ represent numbers of water and organic solvent molecules in the same shell, respectively. It is conceivable that *n*_w_ takes values between 0 and *n*. When *n*_w_ = *n*, 
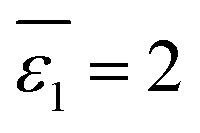
, implying a full first hydration layer on the ion. The ratio of *n*_w_ to *n*_s_ is related to the ratio of bulk molar concentrations of water molecules (*c*_w_) to the given organic solvent molecules (*c*_s_), considering the preferential solvation of an ion in its first solvation layer. This relation is formulated in eqn (7),

7 *n*_w_/*n*_s_ = *Kc*_w_/*c*_s_

in which *K* is the equilibrium constant for preferential solvation of the ion. For convenience, we set *K* to 100 as recommended by Samec *et al.*^32^ The values of *c*_w_ and *c*_s_ for organic solvents saturated with water are calculated and listed in Table S1 in the ESI.† Accordingly, values of *n*_w_ and *n*_s_ for the first solvation layer surrounding the Li^+^ cation (set its hydration number = 4;^33^ see Table S2 in the ESI†) in the varying organic solvents saturated with water are calculated and listed in Table S3 in the ESI† (see the third column). Similarly, *n*_w_ and *n*_s_ data for Cl^−^ anion (set its hydration number = 6; see Table S2 in the ESI†) can also be obtained (see Table S4 in the ESI†).

The unknown radius *b* in eqn (5) can be estimated by eqn (8), in which *V*_1_ is the volume of the first solvation layer, *d*_w_ and *d*_s_ represent the Stear–Eyring diameters (defined in the title of Table S1, ESI†) of water molecules and solvent molecules, respectively. With the Stear–Eyring diameter formula, *d*_w_ equals 0.31 nm, and *d*_s_ of different organic solvent molecules are also calculated and listed in Table S1 in the ESI.†

8 *V*_1_ = *n*_w_π*d*_w_3/6 + *n*_s_π*d*_s_3/6 = (4π/3)(*b*^3^ − *a*^3^)

Now we need to consider the double-layer solvation model of ions in “wet” organic solvents (*e.g.*, aniline and *n*-octanol; see Table S1 in the ESI†). The electrostatic term (in kJ mol^−1^) of the double-layer model is similar to eqn (5) and formulated in eqn (9). In eqn (9), *ε*_1_ equals 2 (*cf.*eqn (6)), corresponding to a full first hydration layer on the ion. And *ε*_2_ equals 29, as recommended by Abraham *et al.*^29^ In this model, we assume that the second solvation shell also consists entirely of water molecules. Hence, *b* – *a* = *c* – *b* = *d*_w_/2 = 0.155 nm.9
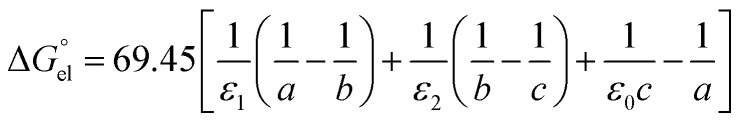


Finally, we return to the calculation of the contribution of the neutral term. It is expressed mathematically in eqn (10).^29^10
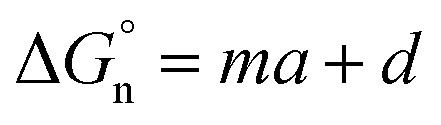


In eqn (10), *m* and *d* are known empirical constants for various solvents. We note that the difference between these two constants in different solvents is within one order of magnitude. Besides, because the neutral term is only a minor contributor to the total Gibbs solvation energy, for simplicity, we used *m* = −8.58 × 10^4^ J mol^−1^ nm^−1^ and *d* = 40.8 × 10^3^ J mol^−1^ estimated for the water in all calculations. This results in 
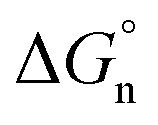
 values of 34.3 kJ mol^−1^ for Li^+^ and 25.27 kJ mol^−1^ for Cl^−^, respectively, as shown in Table S2 in the ESI.†

To summarize: the standard transfer potential of ions with the double-layer hydration model can be calculated using eqn (9), (10), (4), (3), and (1) in sequence; while the standard transfer potential of ions with the monolayer (mixed) solvation model can be calculated using eqn (5), (6), (7), (8), (10), (4), (3), and (1) in sequence.

### 3.2 Two-electrode configuration: with/without aqueous reference phase for the organic phase

In our prior work,^17,18,23,24,34^ we used Ag/AgCl instead of Ag/AgTB as the electrode for the oil phase (which normally contained a supporting electrolyte with TB^−^ as the anion). The main reason is that Ag/AgTB electrode is difficult to prepare, because commercial organic solvents generally contain water (∼26 mM in DCE),^2^ making it very likely that the anodic electrolysis product of an Ag wire is Ag/Ag_2_O rather than AgTB. Besides, TB^−^ is a weakly coordinating anion,^35^ making it difficult to co-precipitate with Ag^+^ to form a stable layer of AgTB. In the present work, we verify the validity of Ag/AgCl employed as the CE/RE which is located within the organic phase directly. Fig. S3 in the ESI† illustrates this two experimental configuration, in which an Ag/AgCl wire is directly placed inside the outer organic phase as depicted in panel A and an identical wire is instead put within an aqueous reference phase that is housed in a larger micropipette contacting with the oil phase, as shown in panel B. At a first glance, an organic reference electrode should have a common anion with the organic supporting electrolyte, *e.g.*, AgTB. However, from a practical viewpoint, selecting an appropriate reference electrode for electrochemical measurements in an organic solution is a generally recognized difficult task (see Section 4.5 in ref. 36). For electrochemistry at the macro-liquid/liquid interface (mm^2^ or cm^2^ scale), a four-electrode potentiostat with *iR* drop compensation is normally employed,^37^ in which the reference potential for the organic phase is realized by a secondary water/organic interface with the interfacial potential fixed by the shared common ion. For example, if the organic phase contains BATB as the supporting electrolyte, the secondary water, *i.e.*, the aqueous reference phase (see Fig. S3B, ESI†), will contain BACl. The potential at the secondary water/organic interface will be poised and given by the Nernst equation for BA^+^ partition equilibrium between the two phases. A silver wire coated with a layer of AgCl can be simply employed as the reference electrode in the secondary water phase connecting with the external power source (*e.g.*, a potentiostat). This quasi-reference electrode composition in the organic phase can be denoted as: Ag/AgCl/BACl/BATB. This Ag/AgCl/BACl/BATB reference electrode was called an oil/water-type reference electrode coined by Senda *et al.*^38^ This oil/water-type reference electrode has been successfully and widely used in the field of four-electrode macro-ITIES electrochemistry.^12^ However, it is applicable for the ITIES of any size, regardless of the dimension of the interfacial area (*vide infra*). Interestingly, Fig. S3B in the ESI† is essentially the same experimental configuration as that used by Taylor and Girault in 1986 to firstly miniaturize an ITIES.^19^ The difference is that our setup in Fig. S3B in the ESI† avoids the use of U-tubes and only requires two easy-to-prepare micropipettes. In fact, for the electrochemistry at the micro- and/or nano-ITIES supported at a micropipette or nanopipette, a two-electrode experimental setup is good enough to get a high-quality voltammogram. Because the capacitive current and *iR* drop can be minimized greatly.

**Fig. 2:**
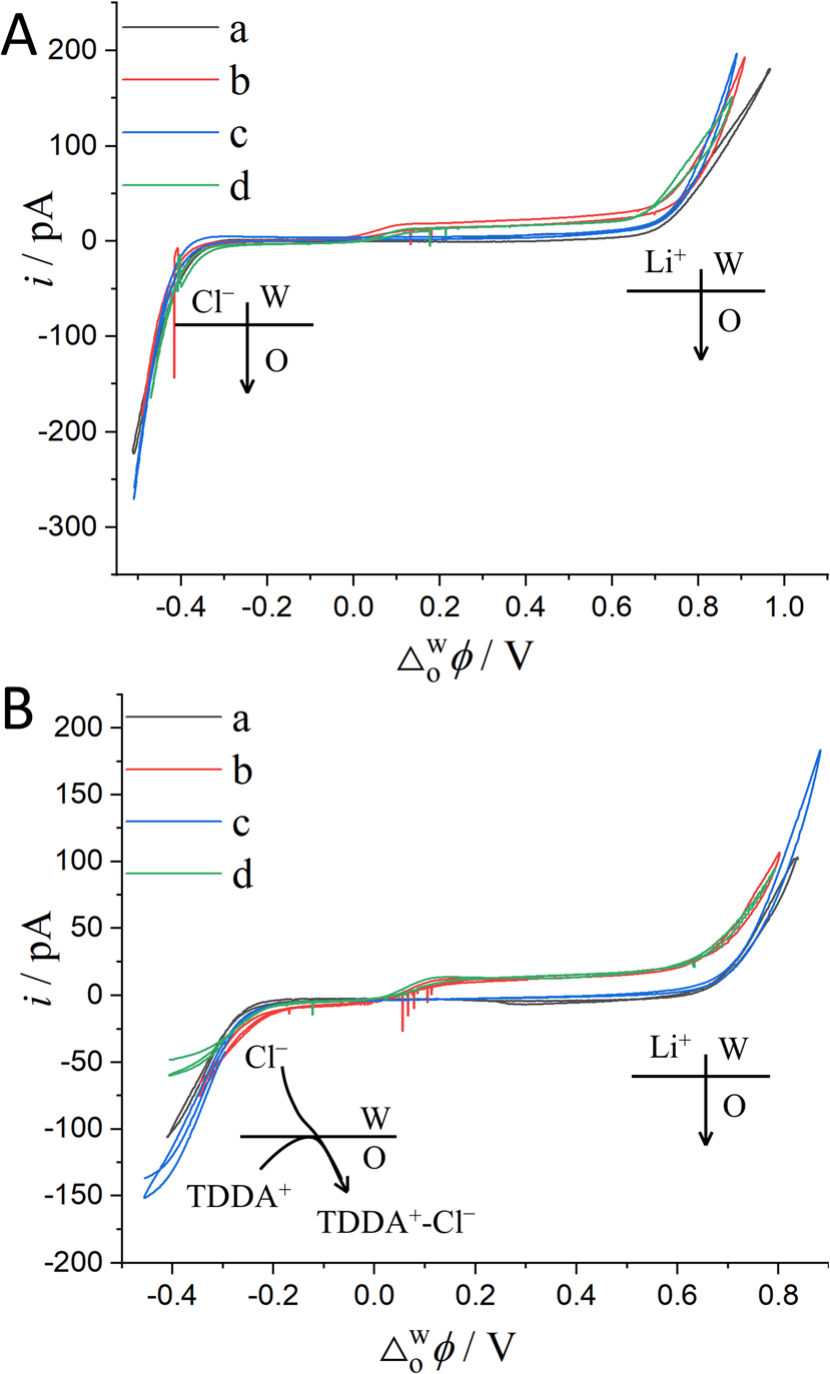
(A) CVs (20 mV s^−1^) recorded at an ITIES formed at the orifice of a micropipette with electrochemical cells 1 and 3 specified in Scheme 1, for traces “a” (the i.d. of pipette is 1.1 μm, oil: TFT) and “b” (the i.d. of pipette is 1.2 μm), respectively. Traces “c” (the i.d. of pipettes filled with 10 mM LiCl and 10 mM BACl are 1.2 and 11.2 μm, respectively) and “d” (the i.d. of pipettes filled with 10 mM LiCl/3 mM TEACl and 10 mM BACl are 1.2 and 20.2 μm, respectively) were obtained using cells 4 and 5 specified in Scheme 1, respectively. The supporting electrolyte in the TFT phase of these four curves is 5 mM BATB. (B) Except that the supporting electrolyte of the TFT phase was 5 mM TDDATB and the diameter of the micropipette was different, the other experimental conditions were the same as those in panel (A). The i.d. of micropipettes are 1.3, 1.2, 1.2 (filled with 10 mM LiCl) and 19.8 (filled with 10 mM BACl), and 1.2 (filled with 10 mM LiCl and 3 mM TEACl) and 14.6 (filled with 10 mM BACl) μm, for traces “a”, “b”, “c”, and “d”, respectively.

The above justification is further proved experimentally by the cyclic voltammograms (CVs) shown in Fig. 2. For a polarizable ITIES between 10 mM LiCl (w) and 5 mM BATB (TFT), the two well-defined blank CVs (black and blue traces) of Fig. 2A illustrate that both the two-electrode setups of Fig. S3† are useful and practical. With TEA^+^ added *in situ* in the aqueous phase as an internal reference ion, a Galvani potential scale with a PPW width of ∼1.1 V (from −0.40 to +0.70 V, here PPW width equals the difference between the onset transfer potentials) is evaluated (Fig. 2A), according to the work reported by Shao and co-workers.^39^Fig. 2B shows the CVs for a polarizable ITIES between 10 mM LiCl (w) and 5 mM TDDATB (TFT). Except for the different cations of the supporting electrolyte in the oil phase, the other experimental conditions were the same as those in Fig. 2A (see its caption). While, a narrower PPW width of ∼0.95 V (from −0.25 to +0.70 V, here again PPW width equals the difference between the onset transfer potentials) is observed. Specifically, the 10 mM LiCl (w)/5 mM TDDATB (TFT) system features a narrower left potential window. This difference can be attributed to the fact that TDDA^+^ facilitates Cl^−^ transfer from w to TFT through the ITIES. It is well recognized that halide salts of TDDA^+^ are often used as cationic surfactants. And hence the positively-charged polar head of TDDA^+^ locates at the water side *via* the adsorption state at the w/TFT interface^40^ and interacts with Cl^−^ more strongly with respect to that between BA^+^ and Cl^−^ across the interface. Very likely, TDDA^+^ shuttles the transfer of Cl^−^ ions from phase w to the phase TFT with the interfacial ion-pair (TDDA^+^–Cl^−^) as the intermediate species.^41^

### 3.3 PPW width *vs.* water content in organic solvents

#### 3.3.1 PPW width *vs.* solubility of water in organic solvents

**Fig. 3:**
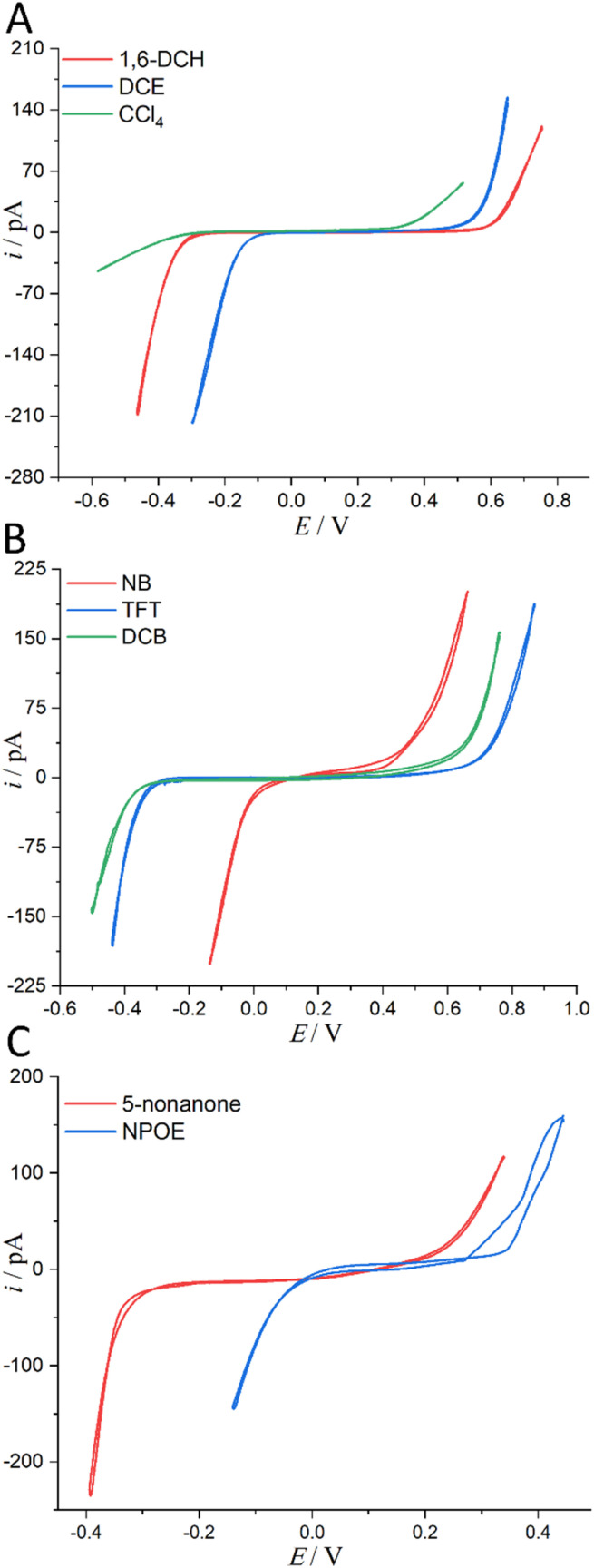
(A) CVs (20 mV s^−1^) recorded at an ITIES separating the aqueous electrolyte (10 mM LiCl) and the organic electrolyte (5 mM BATB or 20 mM TDDATB for CCl_4_ exclusively). More information is detailed in electrochemical cells 1 and 2 in Scheme 1. The i.d. of the orifices of the micropipettes for housing the aqueous phases in contact with either 1,6-DCH (red trace, panel (A)), or DCE (blue trace, panel (A)), or CCl_4_ (green trace, panel (A)), or NB (red trace, panel (B)), or TFT (blue trace, panel (B)), or DCB (green trace, panel (B)), or 5-nonanone (red trace, panel (C)), and or NPOE (blue trace, panel (C)), are all 1.2 μm. Note that the aqueous within the pipette and the adjoining organic solvents were saturated with each other (see step 1 of “Regulation of Water Content in Organic Solvents”, ESI,† for more details).

Considering the toxicity, danger, and inaccessibility of most organic solvents in Table S1 in the ESI,† we decided to use the solubility of water in organic solvents as the value of water content. Experimentally, we used 8 water-saturated organic solvents to form micro-interfaces with organic solvent-saturated water to determine their PPW during polarization (see Fig. 3). In order to be polarized by an external power source, the aqueous electrolyte was 10 mM LiCl (rather than TPrABF_4_, see “Hydrated LiCl *vs.* Non-hydrated TPrABF_4_ as the PPW Probe”, ESI,† for more details), while the oil electrolyte was either 5 mM BATB (seven organic solvents except CCl_4_) or 20 mM TDDATB (for CCl_4_). The experimentally measured widths of PPWs from CVs (Fig. 3) for the ITIESs between 10 mM LiCl (w) and 5 mM BATB in 1,6-DCH, DCE, NB, TFT, DCB, NPOE, and 5-nonanone are 1.055 (Table 2), 0.79 (Table 2), 0.566 (Table S4, ESI), 1.14 (Table S4, ESI), 1.104 (Table S4, ESI), 0.504 (Table S4, ESI), and 0.679 V (Table S6, ESI†), respectively. While the experimental PPW width of the ITIES between 10 mM LiCl (w) and 20 mM TDDATB (CCl_4_) is 0.798 V (see Table 2, last column). The corresponding theoretical values are 1.23, 0.97, 0.49, 1.42, 1.38, 1.0, 0.85, and 2.2 V, for an ITIES between LiCl (w) and 1,6-DCH, or DCE, or NB, or TFT, or DCB, or NPOE, or 5-nonanone, and or CCl_4_, respectively (*cf.*Tables 2, S4 and S6, ESI†). Comparing the PPW theoretical and experimental values of these 8 ITIES, the differences are mostly within 0.3 V. For example, the PPW difference between the theoretical and experimental values for the w/1,6-DCH interface is 0.175 V: the relative error is 16.6% (see Table 2). However, the difference between theoretical and experimental results of non-polar/low-polar organic solvents (*ε*_0_ ≤ 5, *e.g.*, CCl_4_, toluene, or TCM) and hydrogen-bonded organic solvents (*e.g.*, NPOE or 5-nonanone) is quite large. For example, when CCl_4_ is used as the organic solvent, the PPW width difference (theory *vs.* experiment) is about 1.4 V. This dramatic difference can be explained by two reasons: (1) apolar molecule like CCl_4_ is not a good solvator to solvate ions, thus invalidating the modified Born model; and (2) ionic liquid like TDDATB employed as the supporting electrolyte in CCl_4_ for the PPW measurement is not a “normal” electrolyte.^42^ In a word, sometimes simply comparing theoretical and experimental values is not very meaningful. Some arguments can be summarized as follows: (1) the theoretical model in this work is a modified Born ion solvation model. The original version of this model is that ions of a given radius are immersed in continuous dielectric solvent molecules; this model does not consider the structural details and interactions at the molecular/atomic level taking emergence of the water finger^13–16^ as an example. (2) There are various experimental measurement methods: for example, this work uses ion polarizable ITIES^43^ to measure the PPW width; hence, the choice of supporting electrolyte in the two phases is critical, *e.g.*, the use of TDDATB in the oil phase leads to a narrowing of the PPW (see Fig. 2). (3) Theoretical models often ignore some details of the experimental methods, such as the ion-pair formation mechanism of ion transfer^41^ in this work, the possible formation of ionosomes^17,18,23^ in the organic phase, *etc.* In fact, with a three-phase electrode configuration which features two polarizable interfaces,^44^ the experimental PPW width (0.97 V, see Table S4, ESI†) at the w/NPOE interface matches perfectly with the theoretical value (1.0 V, see Table S4, ESI†). Of course, the above results show that: (1) the ion solvation model needs to be improved; and (2) the experimental method for PPW measurement also needs to be further developed.

**Table 1:** Comparison between the theoretical standard Gibbs free energy 
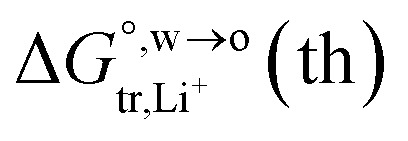
 of Li^+^ (*a* = 0.078 nm) transferred from water (w) to organic solvents (o) and the experimental value 
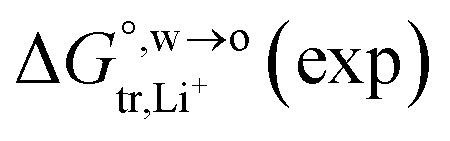
, where 
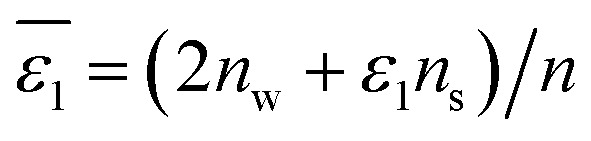

Solvent	*c*_w_ (mol L^−1^)	*n*_w_(*n*_s_)	*b* (nm)	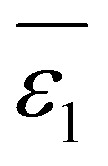	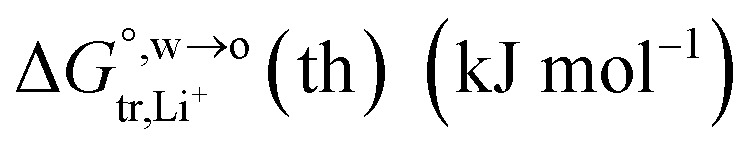	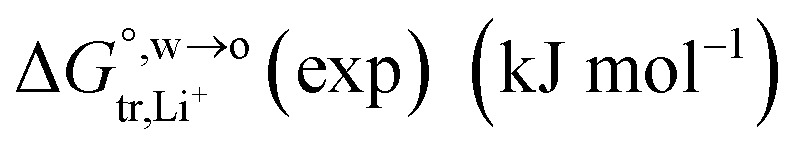
DCE	0.11	1.9(2.1)	0.349	2.05	45	53^a^
DCM	0.111	1.7(2.3)	0.334	2.03	49	55.8^b^
1,4-DCBu	0.076	1.9(2.1)	0.383	2.06	52	57^a^
1,6-DCH	0.057	1.8(2.2)	0.42	2.07	58	60^a^
Nitroethane	0.841	3.5(0.5)	0.277	1.99	18	—
1-Nitropropane	0.333	3.0(1.0)	0.311	1.99	33	—
2-Nitropropane	0.276	2.9(1.1)	0.317	1.98	36	—
TCM	0.063	1.3(2.7)	0.37	2.07	67	—
CCl_4_	0.0077	0.3(3.7)	0.423	2.07	104	—

a Ref. 45.

b Present study.

**Table 2:** Comparison of the theoretical standard Gibbs free energy 
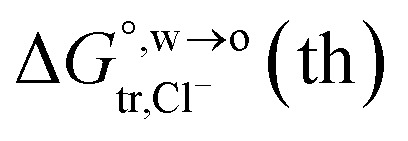
 of Cl^−^ (*a* = 0.181 nm) transferred from water (w) to organic solvents (o) and the experimental value 
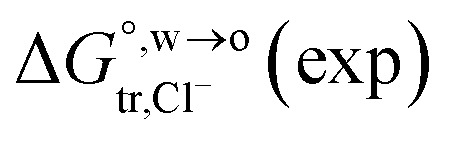
, where 
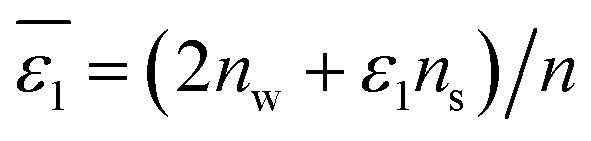
. Note that the last two columns (from the left) are theoretical (Δ*ϕ*(th)) and experimental (Δ*ϕ*(exp)) values of the PPW width of LiCl at the w/o interfaces, in which the potential scale of Li^+^ transfer at w/o is converted with the data of standard Gibbs free energy of Li^+^ transfer as listed in Table 1. The experimental PPWs in the present work are shown in parentheses in the last column

Solvent	*c*_w_ (mol L^−1^)	*n*_w_(*n*_s_)	*b* (nm)	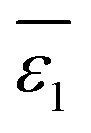	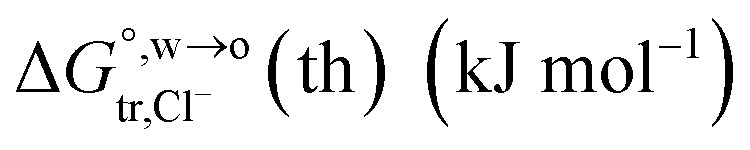	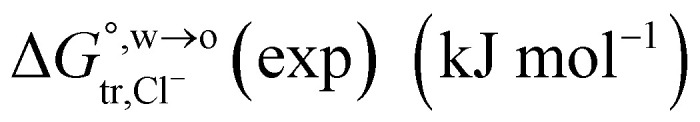	Δ*ϕ*(LiCl,th) (V)	Δ*ϕ*(LiCl,exp) (V)
DCE	0.11	2.8(3.2)	0.410	2.05	49	38^a^	0.97	0.94(0.79)
DCM	0.111	2.5(3.5)	0.395	2.03	51	46.4^b^	1.04	1.06
1,4-DCBu	0.076	2.8(3.2)	0.449	2.06	56	46^a^	1.12	1.07
1,6-DCH	0.057	2.7(3.3)	0.488	2.07	61	47^a^	1.23	1.11(1.055)
Nitroethane	0.841	5.2(0.8)	0.335	1.99	24	28.7^c^	0.44	—
1-Nitropropane	0.333	4.5(1.5)	0.370	1.99	34	—	0.69	—
2-Nitropropane	0.276	4.3(1.7)	0.377	1.98	35	—	0.74	—
TCM	0.063	2.0(4.0)	0.432	2.07	69	40^d^	1.41	—
CCl_4_	0.0077	0.4(5.6)	0.493	2.07	105	—	2.2	(0.798)

a Ref. 45.

b Ref. 46.

c Ref. 47.

d Ref. 48.

As mentioned briefly in the Introduction that a negative linear relationship exists between PPW at an ITIES and water content (*c*_w_) in the organic solvents as shown in Fig. S1 in the ESI.† But, note that this figure shows a mixture of three types of organic solvents: halogenated hydrocarbons, aromatic hydrocarbons, and aliphatic ketones. We speculate that in a larger range of ITIES consisting of the same type of organic solvents and water, there may be other more precise quantitative relationships between these two quantities. In fact, we derived that the PPW width is proportional to the negative natural logarithm of the water content of the organic solvents (*i.e.*, –ln *c*_w_; see eqn (11)).

11 PPW (in V) ∝ −ln *c*_w_

**Fig. 4:**
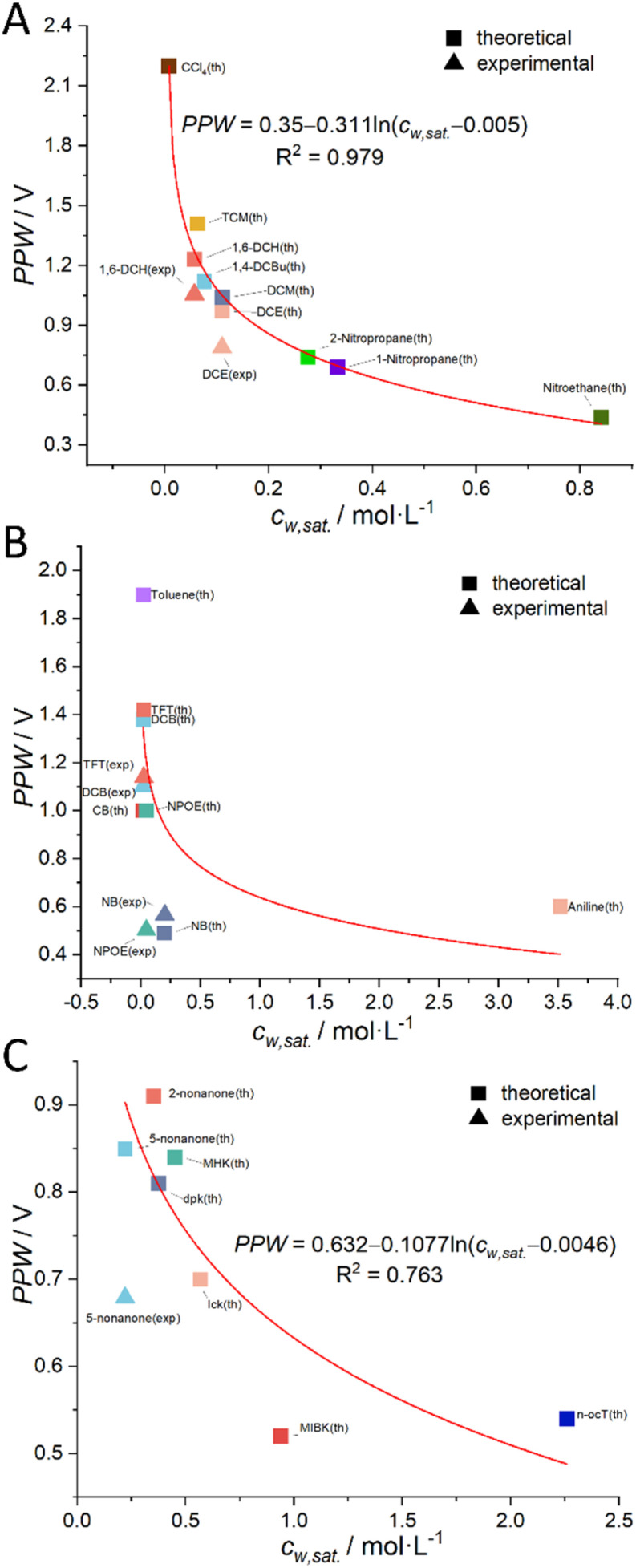
The relationship between the theoretical (solid square) or experimental (solid triangle) PPW width at various water (10 mM LiCl)/oil (5 mM BATB or 20 mM TDDATB) interfaces and the solubility of water in organic solvents (*c*_w,sat._). Note that, in panel (A), the organic solvent employed is a halogenated hydrocarbon; in panel (B), an aromatic hydrocarbon is utilized as the organic solvent; while in panel (C), a ketone or alcohol serves as the organic solvent. In the same panel, one color represents one organic solvent. The seven solid triangles are experimental data from Fig. 3 (CCl_4_ data is discarded), while the remaining data points are theoretical values (listed in Tables 2, S4 and S6, ESI†).

More details can be found in the section “Derivation of the Relation between the PPW Width and Water Content in Organic Solvents” in the ESI.† Following this idea, we classified organic solvents into three categories (see caption of Fig. 4) and plotted Fig. 4 using 7 experimental data from Fig. 3 (experimental data of CCl_4_ is discarded) and 23 (*i.e.*, the total number of organic solvents, see Table S1, ESI†) theoretical values (listed in Tables 2, S4 and S6, ESI†). The best fit equation of Fig. 4A shows that the PPW width is indeed proportional to –ln *c*_w_, as expected. Since the data points in Fig. 4B are more scattered, no fitting analysis was performed. The data points in Fig. 4C do not fit as well as those in Fig. 4A (compare the R^2^ of the two). Fig. 4A and B identify the organic solvents (except aniline) as “dry”, given the small values of *n*_w_. While in Fig. 4C, both single-layer and bilayer models were employed to calculate the theoretical PPW width. It is proved that when the bilayer model is used, the theoretical values are closer to the experimental values, indicating a complete hydration of ions in these “wet” organic solvents. It also suggests that it is not straightforward to determine whether ions are fully hydrated solely based on the water content in organic solvents.

#### 3.3.2 PPW width *vs.* water content of a given organic solvent

**Fig. 5:**
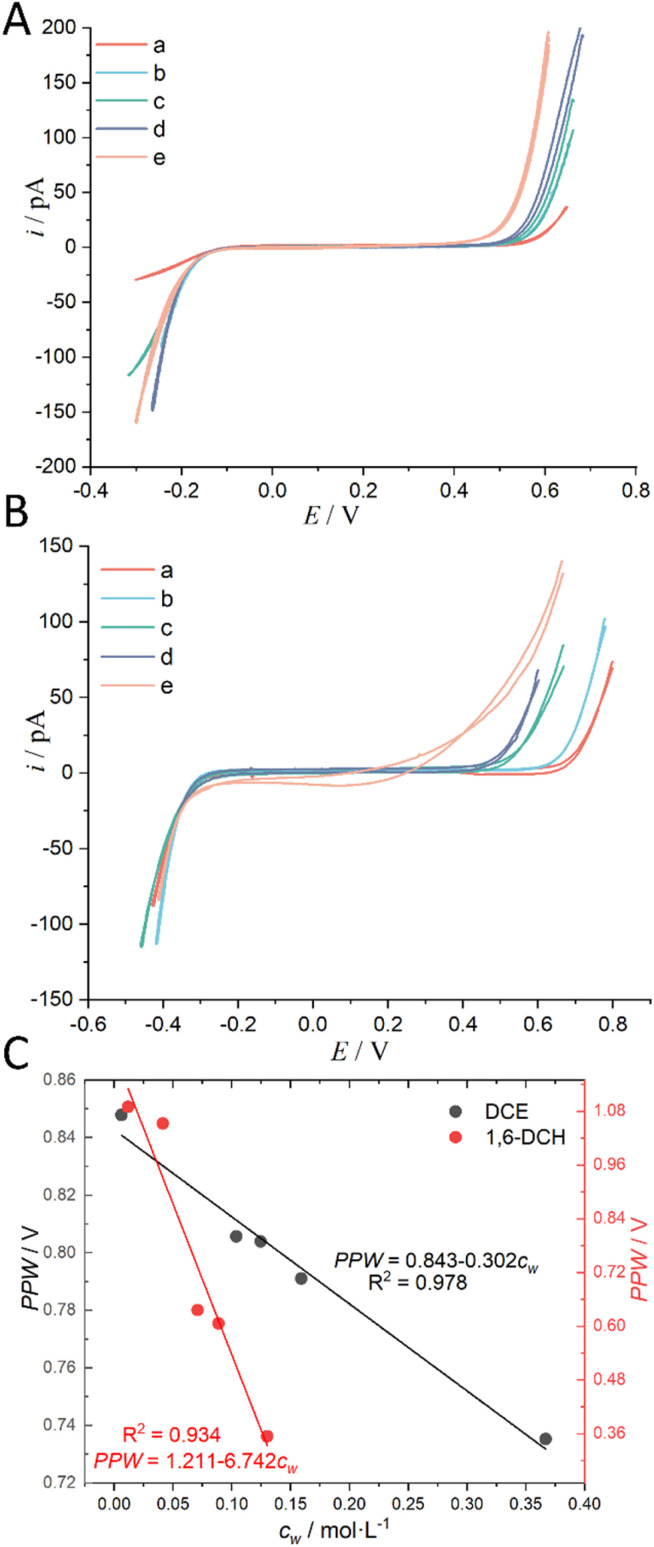
(A) CVs (20 mV s^−1^) recorded at an ITIES between the aqueous electrolyte (10 mM LiCl) and the DCE electrolyte (5 mM BATB) with different water concentrations (“a” ultra-dry DCE, *i.e.*, *c*_w_: 0.00623 mol L^−1^; “b” *c*_w_: 0.104 mol L^−1^; “c” *c*_w_: 0.1246 mol L^−1^; “d” *c*_w_: 0.159 mol L^−1^; and “e” water-saturated DCE, *i.e.*, *c*_w_: 0.367 mol L^−1^). More information is detailed in electrochemical cell 1 in the Scheme 1. The i.d. of the orifices of the micropipettes for housing the aqueous electrolytes for traces “a”–“e” are 1.1, 1.1, 1.1, 1.2, and 1.2 μm, respectively. (B) CVs (20 mV s^−1^) recorded at an ITIES between the aqueous electrolyte (10 mM LiCl) and the 1,6-DCH electrolyte (5 mM BATB) with different water concentrations (“a” ultra-dry 1,6-DCH, *i.e.*, *c*_w_: 0.012 mol L^−1^; “b” *c*_w_: 0.041 mol L^−1^; “c” *c*_w_: 0.071 mol L^−1^; “d” *c*_w_: 0.089 mol L^−1^; and “e” water-saturated 1,6-DCH, *i.e.*, *c*_w_: 0.13 mol L^−1^). More information is detailed in electrochemical cell 1 in the Scheme 1. The i.d. of the orifices of the micropipettes for housing the aqueous electrolytes for traces “a”–“e” are 1.1, 1.1, 1.1, 1.1, and 1.2 μm, respectively. Note that, in order to better compare the CVs, we moved the negative ends of the five CVs in panels (A) and (B) to similar potential locations. (C) The relationship between the PPW width measured at different ITIES (see panels (A) and (B)) and the water concentration (*c*_w_) in the organic solvents. The black and red circles represent data using DCE and 1,6-DCH as the organic solvents, respectively. On *c*_w_, see “Regulation of Water Content in Organic Solvents”, ESI,† for more details.

The above analysis is based on a rough classification of organic solvents. We further used a certain organic solvent (DCE and 1,6-DCH) and introduced a water concentration gradient in it, and performed CV measurements at the micro-ITIES of w (10 mM LiCl)/o (5 mM BATB) to explore the quantitative relationship between its PPW width and the water concentration (*c*_w_) in the organic solvent. The results are shown in Fig. 5 (experimental details are shown in the caption of Fig. 5). Fig. 5A and B both show that the PPW width narrows gradually with the increase of water concentration in the organic solvent, and the change rate of 1,6-DCH is greater than that of DCE. This behavior is quantitatively shown in the slope of the linear relationship between PPW width and *c*_w_ in Fig. 5C. The largest *c*_w_ in Fig. 5C is the solubility of water in the organic solvent, which is measured by us. We stirred the two phases for 24 h to saturate the water in the organic solvent, and measured the water content after standing for 24 h (refer to “Regulation of Water Content in Organic Solvents”, ESI,† for more details). We found that the result was much higher than the literature value (see Table S1, ESI†). The stirring method can lead to emulsification and solubilization, that is, water is in a supersaturated state. Even so, the narrowing of the PPW width with increasing water content was confirmed again. Fig. S7 in the ESI† is a replica of Fig. 5C, except that the data for organic solvents (over)saturated with water have been removed. It is very likely that this linear range (of Fig. S7, ESI†) is located on the linear branch of the low-concentration range of the negative natural logarithmic curve in Fig. 4, and when the water concentration, *c*_w_, reaches a threshold, the PPW width tends to level off. Fig. S8 in the ESI† shows that the increase in water concentration in the oil phase caused by the entry of Li^+^ and Cl^−^ ions into the oil phase under an electric field is negligible and correctable. This experiment also once again confirms the correctness of the concept of water finger which underpins the working mechanism of our proposed method.

## 4. Conclusions

In summary, we employed hydrated Li^+^ and Cl^−^ as probes to attempt to correlate their transfer PPW width at an ITIES and the water content in organic solvents. A hybrid modified Born ionic solvation model was used to calculate the theoretical PPW of LiCl at a series of ITIES, which reconciles with the experimental results fairly. New insights from both experimental and theoretical perspectives point to the essential role of water content in organic solutions. Briefly, the PPW width is proportional to the negative natural logarithm of the water content (in a wide concentration range) in the organic media. While the PPW width has a negative linear relationship with the water content over a small concentration range. This work demonstrates that ion transfer voltammetry, a weak-interaction electrochemical method, can conveniently measure the water content in organic solvents, which will be beneficial to fields such as analytical chemistry, physical chemistry, and synthetic chemistry in solution. This work also points out a direction for future theoretical research: the effect of water in organic solvents on water finger mechanism during ion transfer at an ITIES needs to be seriously considered.

## Data availability

All data underlying this article have been included in the main text and ESI.†

## Author contributions

Conceptualization: H. Deng; funding acquisition and supervision: H. Deng and X. Sun; conducting experiments and data analyses: S. Jin, L. Yang, S. He, and T. Fang; conducting auxiliary experiments and data analyses: D. Cai, Q. Hu, and X. Huang; writing: original manuscript by S. Jin, revised manuscript by H. Deng and X. Sun. All authors contributed to writing the manuscript and to the discussions.

## Conflicts of interest

There are no conflicts to declare.

## Supplementary Material

SC-016-D5SC00527B-s001
