# Supplementary material for: Polarizable potential window at soft molecular interfaces as a quantitative descriptor for the water content in organic solvents
Source: Chem Sci. 2025 Feb 18;16(12):5017–27. doi: 10.1039/d5sc00527b (PMC11833678; doi:10.1039/d5sc00527b)
Supplement: SC-016-D5SC00527B-s001 [file SC-016-D5SC00527B-s001.pdf]

Supplementary Information (SI) for Chemical Science.
This journal is © The Royal Society of Chemistry 2025

Electronic Supplementary Information

Polarizable Potential Window at Soft Molecular Interfaces as a Quantitative Descriptor for the Water Content in Organic Solvents

Siqi Jin,[a] Lifang Yang,[a] Sijia He,[a] Taoxiong Fang,[a] Xiaohang Sun,*[a] Dandan Cai,[b] Qiong Hu,[c] Xinjian Huang,[d] and Haiqiang Deng*[a]

[a]School of Chemical Engineering and Technology, Sun Yat-sen University, Zhuhai 519082, China. E-mails: denghq9@mail.sysu.edu.cn; sunxh25@mail.sysu.edu.cn

[b]National Engineering Research Center for Carbohydrate Synthesis, School of Chemical Engineering, Jiangxi Normal University, Nanchang 330022 China

[c]Guangxi Key Laboratory of Agricultural Resources Chemistry and Biotechnology, College of Chemistry and Food Science, Yulin Normal University, Yulin 537000, China

[d]Institute of Intelligent Technology, Midea Corporate Research Center, Foshan 528311, China

Ag | AgCl | 5 mM TEACl, 10 mM LiCl (*aq* in pipette) | 5 mM BATB (TFT) | AgCl | Ag (cell s1)

Ag | AgCl | 10 mM $TPrABF_4$ or 10 mM LiCl (*aq* in pipette) | 5 mM BATB (TFT, DCB, DCE, NB, NPOE, 5-nonanone, 1,6-DCH) | AgCl | Ag (cell s2)

Ag | AgCl | 10 mM $TPrABF_4$ or 10 mM LiCl (*aq* in pipette) | 20 mM TDDATB (CCl_4) | AgCl | Ag (cell s3)

Scheme S1. Schematic depiction of the electrochemical cell composition used in the two-electrode micropipette ion-transfer voltammetry. TEACl: tetraethylammonium chloride; $TPrABF_4$: tetrapropylammonium tetrafluoroborate; BATB: bis(triphenylphosphoranylidene) ammonium tetrakis(pentafluorophenyl)borate; TDDATB: tetradodecylammonium tetrakis(pentafluorophenyl)borate; TFT: α,α,α-trifluorotoluene; DCB: 1,2-dichlorobenzene; DCE: 1,2-dichloroethane; NB: nitrobenzene; NPOE: 2-nitrobenzene octyl ether; 1,6-DCH: 1,6-dichlorohexane; and CCl_4: carbon tetrachloride. Note: water and organic solvents were not saturated with each other. For CV measurements, the volume of organic solvent was always 5 mL.

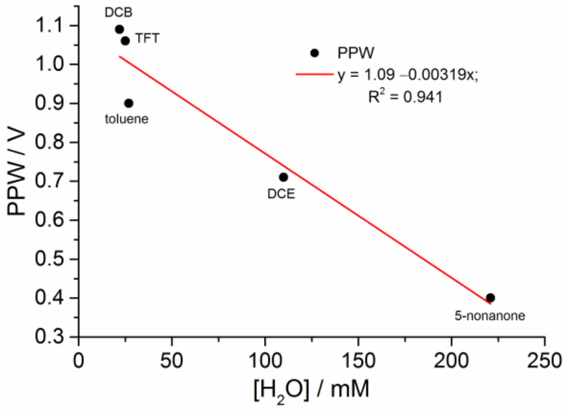

Figure S1. Correlation of the polarizable potential window (PPW) at interfaces between water and different organic solvents and the solubility/content of water in the organic solvents. Note that herein the PPW denotes simply the difference between the onset transfer potentials for Li^+ and Cl^- at any interface between two immiscible electrolyte solutions (ITIES), respectively.

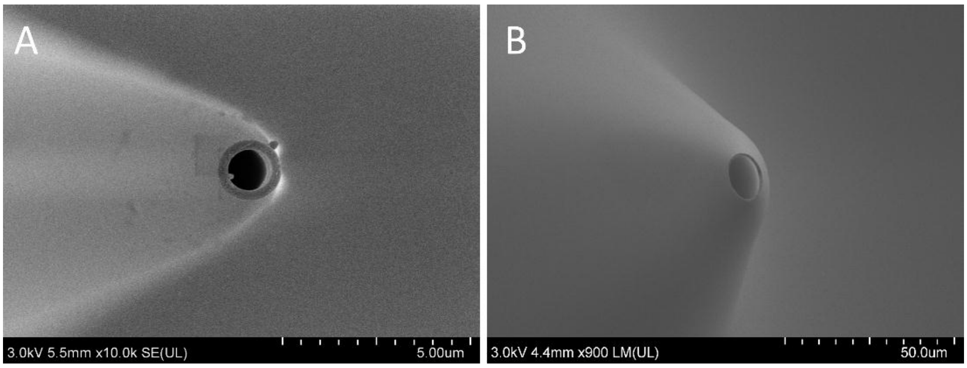

Figure S2. SEM images of (A) a typical 1.2 μm inner diameter (i.d.) borosilicate glass and (B) a typical 11.5 μm i.d. quartz glass micropipettes pulled by a PC-100 puller and a P-2000 puller, respectively.

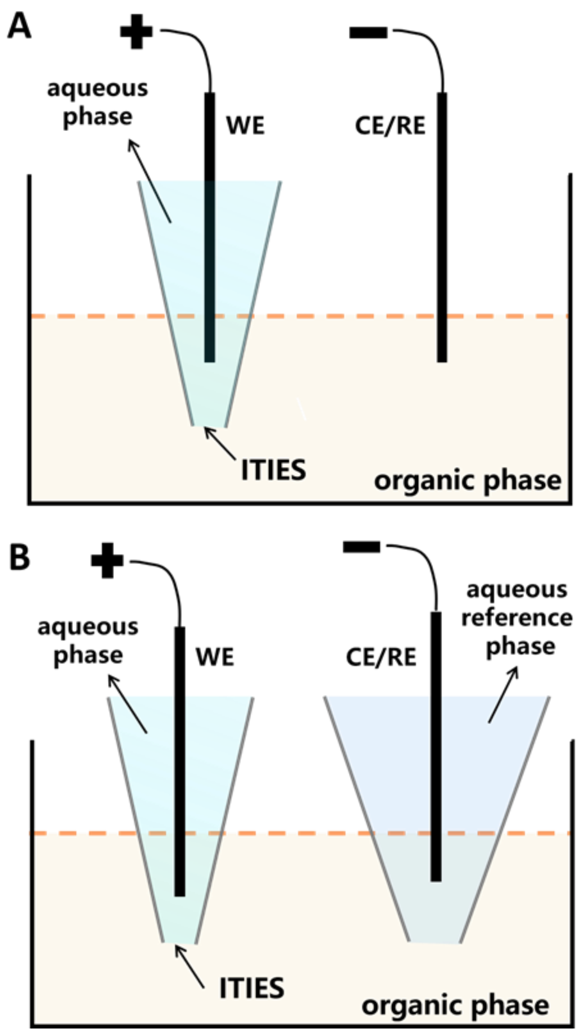

Figure S3. (A) Schematic diagram of the two-electrode cell with the working electrode (WE, an Ag/AgCl wire) housed inside the aqueous-phase-filled micropipette and the counter/reference electrode (CE/RE, an Ag/AgCl wire) inside the outer organic phase. (B) Schematic diagram of the two-electrode cell in a similar configuration as in panel A but with an aqueous reference phase (housed in a larger micropipette) for the oil phase for a better-defined potential control at the CE/RE. The ITIES forms at the orifice of the micropipette that is adjacent to the organic phase. Note that the positive bias of the aqueous phase with respect to the organic phase is shown just as an example, as cyclic voltammogram (CV) is a potential sweep method.

Experimental PPW Width Protocol.

As shown in Figure S4A, the PPW width at an ITIES equals the difference of onset transfer potentials for Li^+ and Cl^-, plus 2 × 0.052 V + 2 × 0.023 V (i.e., plus 0.15 V), in which 0.052 V (an averaged empirical value from multiple CV measurements by us, see e.g., Figure S4B) refers to the difference between half-wave transfer potential with respect to the onset transfer potential, and 0.023 V points to the difference between standard transfer potential and half-wave transfer potential (see Table 2 in ref.[1]). Note that ion transfer at an ITIES is almost always a Nernstian process.

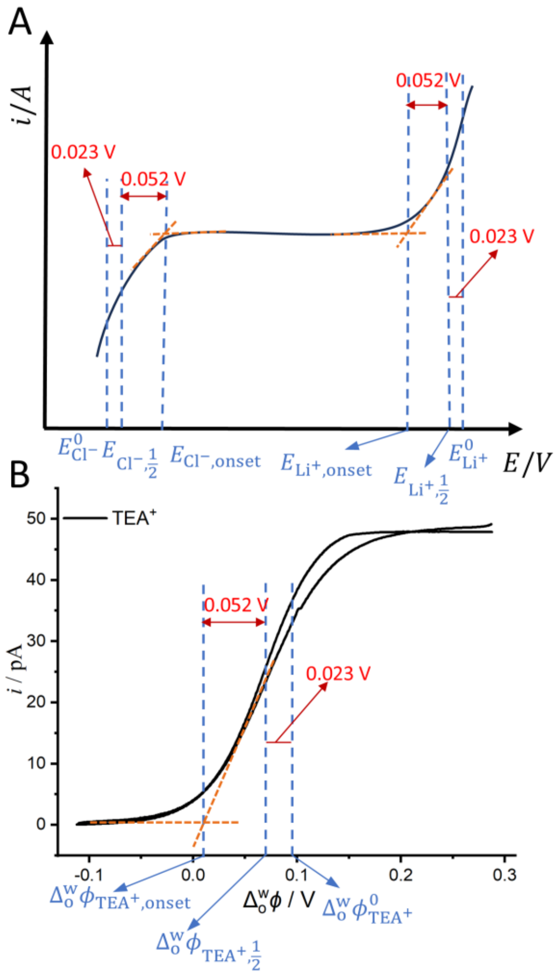

Figure S4. (A) Schematic for the relationship between standard ion transfer potential, half-wave ion transfer potential and onset ion transfer potential. (B) CV obtained within the exclusive TEA^+ transfer potential range (from −0.11 to 0.29 V). The potential of this CV has been converted to the Galvani potential scale with the half-wave transfer potential of TEA^+ being 0.076 V at the water/TFT interface, according to the work of Shao and coworkers.[1] The aqueous electrolyte used was 5 mM TEACl and 10 mM LiCl, with 5 mM BATB as the organic electrolyte dissolved in TFT. The CV was conducted at the water/TFT interface, supported at a micropipette with an i.d. of 1.2 µm. The CV experiment was conducted in a two-electrode system, where two Ag/AgCl wires were inserted into the water and TFT phases, respectively, and connected to the potentiostat. More details are shown as cell s1 in Scheme S1. The scan rate was 20 mV/s.

Hydrated LiCl vs. Non-hydrated $TPrABF_4$ as the PPW Probe.

We used hydrated LiCl and non-hydrated $TPrABF_4$ as probe ions of the aqueous phase, respectively, and formed micro-ITIES with the same eight organic solvents (commercial, not saturated with water) containing BATB or TDDATB as the supporting electrolyte as in Figure 3 of the main text, and carried out CV measurements. The results are shown in Figures S5 and S6.

We observed the following phenomena:

1. Using LiCl as the probe ions: with increasing water content in the organic solvent, the PPW width significantly narrows (e.g., TFT vs. DCE, see Figure S6), showing a clear and consistent trend.
2. Using $TPrABF_4$ as the probe ions: The PPW width also shows a trend similar to that of LiCl, but with a smaller degree of variation (see Figure S6) and larger errors (compared to LiCl, the PPW width for an identical ITIES is narrower and measurement errors are more pronounced).
3. Regardless of the organic solvent, the PPW obtained with LiCl as the probe is always wider than that obtained using $TPrABF_4$ as the probe.
4. Generally, when $TPrABF_4$ is used as the probe ions, the CV has a positive current offset.

The first three points mentioned above indicate that non-hydrated large organic complex ions ($TPrA^+$ and BF_4^-) are less sensitive to the water content in organic solvents. This may be because large organic complex ions such as $TPrA^+$ are more likely to interact with the dominant organic molecules with lower polarity and higher entropy (vs. H_2O) after crossing the interface, thus causing their PPW to narrow. The fourth point aforementioned is because a considerable amount of $TPrABF_4$ has been distributed into the oil phase during CV measurement, while LiCl does not have such behavior.

Therefore, we selected hydrated LiCl as a probe to study the relationship between PPW width and water content in organic solvents.

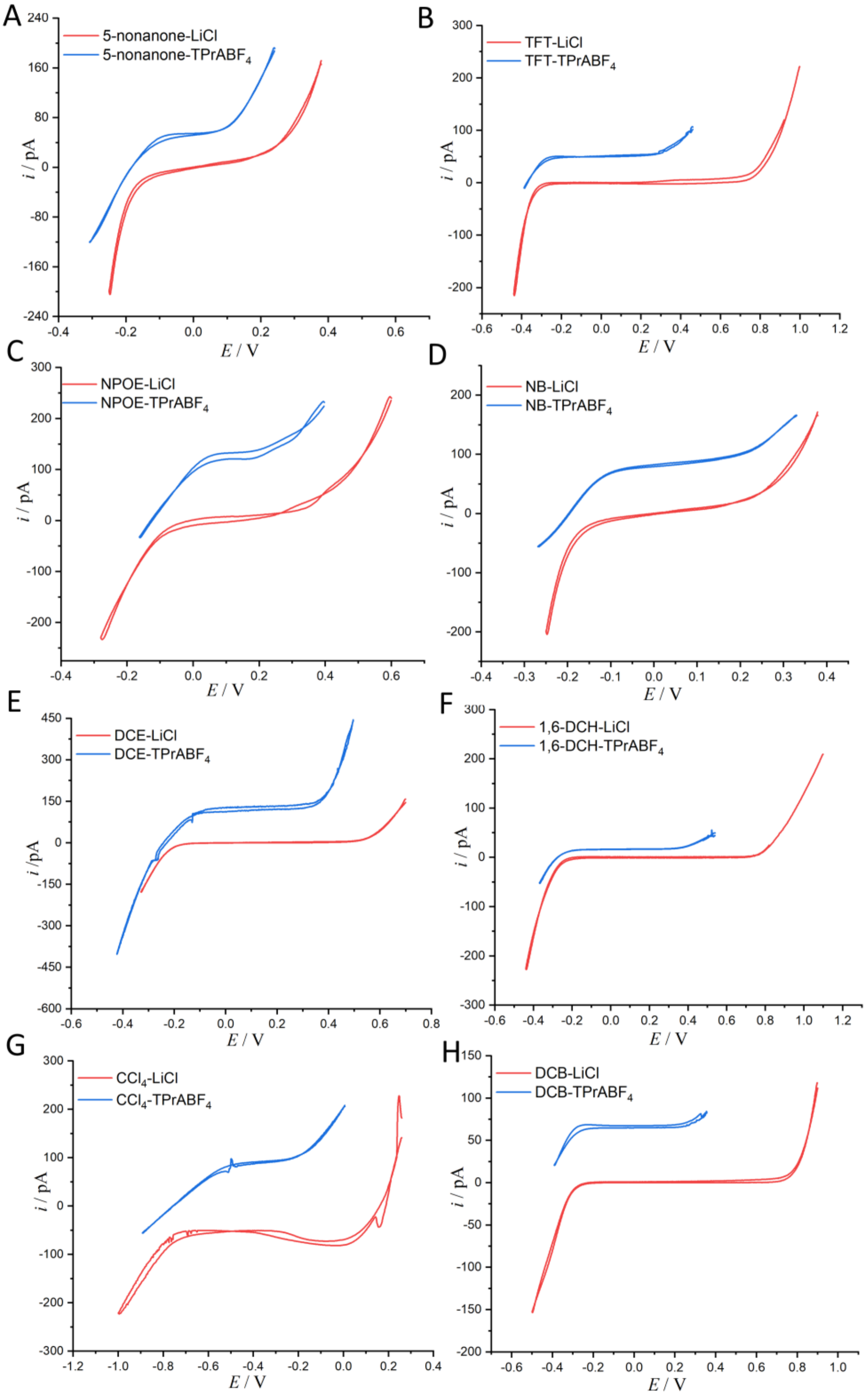

Figure S5. CVs (20 mV/s, potential not calibrated to Galvani scale) recorded at a range of different ITIES, in which the aqueous electrolyte was either 10 mM LiCl (red trace) or 10 mM $TPrABF_4$ (blue trace), and the organic electrolyte was either 5 mM BATB (see cell s2 in Scheme S1 for more details) or 20mM TDDATB (used exclusively for CCl_4, see cell s3 in Scheme S1 for more details). When LiCl was used as the aqueous electrolyte, the i.d. of the orifices of the micropipettes housing the aqueous phases in contact with 5-nonanone (panel A), TFT (panel B), NPOE (panel C), NB (panel D), DCE (panel E), 1,6-DCH (panel F), CCl_4 (panel G), and DCB (panel H) were 4.4, 1.2,

1.1, 4.4, 1.0, 1.3, 1.3, and 1.2 µm, respectively. When $TPrABF_4$ was used as the aqueous electrolyte, the i.d. of the corresponding micropipette orifices were 1.1, 1.1, 1.2, 1.1, 1.1, 1.1, 1.1, and 1.1 µm, respectively. Note that, in order to better compare the CVs, we moved the negative ends of the two CVs to similar potential locations. Note: water and organic solvents were not saturated with each other.

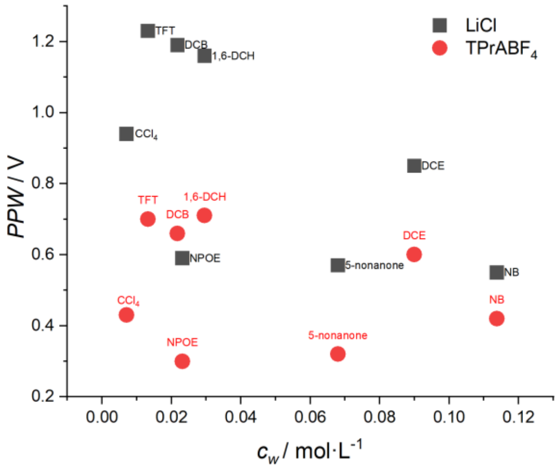

Figure S6. The relationship between the PPW width measured at various ITIES (see Figure S5) and the water concentration (c_W, as measured by Karl Fischer titration, Metrohm 852 + 860) in the organic solvents. The black solid squares and red solid circles represent data for LiCl and $TPrABF_4$ being used as the aqueous supporting electrolyte, respectively.

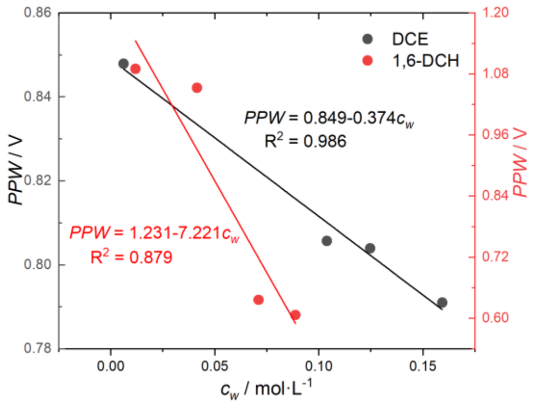

Figure S7. The relationship between the PPW width measured at different ITIES (see panels A and B of Figure 5 in the main text) and the water concentration (c_W) in the organic solvents. The black and red circles represent data using DCE and 1,6-DCH as the organic solvents, respectively. On c_W, refer to "Regulation of Water Content in Organic Solvents" (see below), for more details. Unlike Figure 5C of the main text, data for water-saturated organic solvents are not included here.

Changes in Water Volume within the Micropipette before and after CV Measurements and its Effect on the Water Content in the Organic Solvents.

When the aqueous phase contacts the organic phase, water molecules tend to enter the organic phase. When performing CV measurements, hydrophilic Li^+ and Cl^- will also carry water molecules when entering the organic phase. Therefore, in theory, our method may cause positive errors. In order to explore its actual impact, we selected DCE with a moderate water content as the organic solvent and performed a CV measurement (see caption of Figure S8 for experimental details). The experimental results show that this effect is insignificant/negligible. The specific calculation is as follows:

$V(H_2O) = \pi r^2 h = 3.1416 \times (0.58/2)^2 \times 1.1274 = 0.298\ mm^3 = 2.98 \times 10^{-7}$ L

As a first approximation, pure water is used instead of dilute LiCl (10 mM) aqueous solution to calculate the water concentration in DCE phase.

$n(H_2O) = 55.5$ mol/L $\times 2.98 \times 10^{-7}$ L $= 1.65 \times 10^{-5}$ mol

$c(H_2O) = n(H_2O)/[V(H_2O) + 5\ mL] = 3.30 \times 10^{-3}$ mol/L = 3.30 mM

While the water content in the commercial DCE employed in our experiment is 1300 ppm (90 mM), as determined by the Karl Fischer titration (Metrohm, model: 852 + 860).

Hence, the positive relative error: 3.30/90 × 100% = 3.67%.

In fact, this experiment proves that Li^+ and Cl^- migrate into the organic phase (under the action of electric field) through the formation of ion-water finger complexes, thereby validating the working mechanism of our proposed measurement method.

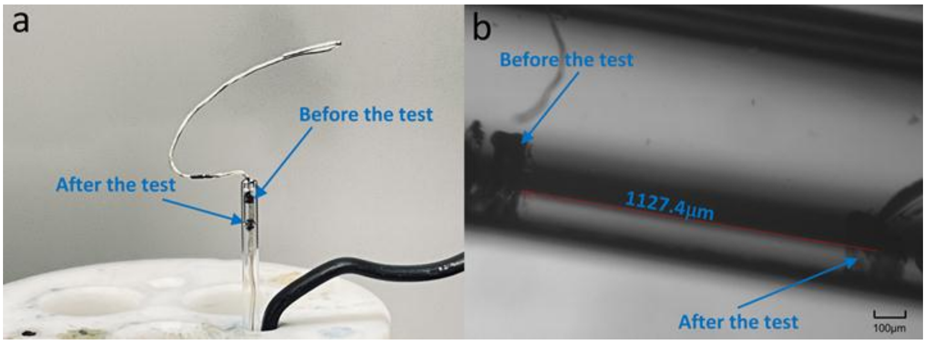

Figure S8. (a) Change in the liquid level (marked by black line) within the glass micropipette before and after one-cycle CV (20 mV/s) measurement, in which the aqueous electrolyte was 10 mM LiCl filled within a glass micropipette with an i.d. of 1.2 μm, and the organic electrolyte was 5 mM BATB in 5 mL DCE (see cell s2 in Scheme S1 for more details); and (b) Zoom-in view of the liquid level change (see panel a) observed under optical microscope.

Regulation of Water Content in Organic Solvents.

(1) Prepare organic solvents saturated with water. Place equal volumes of Millipore water (e.g. 10 mL) and organic solvent (e.g. 10 mL) in a glass vial, and place the vial on a magnetic stirrer for 24 hours to allow the solvents to reach mutual saturation. Then, let the solvent mixture stand for 24 hours, and after stratification, take the upper (or lower) aqueous phase saturated with organic solvent and the lower (or upper) organic phase saturated with water for later use. Note that the water contents of water-saturated DCE and 1,6-DCH, measured by Karl Fischer titration (Metrohm, model: 852 + 860), were 5300 ppm (0.367 mol/L) and 2200 ppm (0.13 mol/L), respectively.

(2) Prepare ultra-dry organic solvents. The 3Å molecular sieves (sourced from Macklin) were treated in a Muffle furnace (Hefei Kejing, model: KSL-1200X-M (27L)) at 300 degrees Celsius for 24 hours, and then added to the commercial DCE and 1,6-DCH solvents, respectively, and after standing for 24 hours, the corresponding ultra-dry organic solvents were obtained.[2] The water contents of ultra-dry DCE and ultra-dry 1,6-DCH, measured by the Karl Fischer titration, were 90 ppm (0.00623 mol/L) and 200 ppm (0.012 mol/L), respectively.

(3) Prepare organic solvents of intermediate water concentrations. Add 10 mL of ultra-dry DCE or 1,6-DCH prepared in step 2 to a separatory funnel, and add an equal volume of Millipore water (i.e., 10 mL) on top. The water content in DCE or 1,6-DCH was adjusted by allowing the two phases to be in contact and stand for different periods of time. Then separate the two phases. Note: when taking out the lower organic solvent, generally a little volume (ca. 1 mL) should be left at the end to avoid disturbing the upper aqueous phase. The water content in DCE after standing for 10 minutes, 20 minutes, and 30 minutes, measured by the Karl Fischer titration, was 1500 ppm (0.104 mol/L), 1800 ppm (0.1246 mol/L), and 2300 ppm (0.159 mol/L), respectively; similarly, the water content in 1,6-DCH after standing for 20 minutes, 30 minutes, and 40 minutes was 700 ppm (0.041 mol/L), 1200 ppm (0.071 mol/L), and 1500 ppm (0.089 mol/L), respectively.

Derivation of the Relation between the PPW Width and Water Content in Organic Solvents.

The standard Gibbs energy of *i* transfer from aqueous to organic phase is expressed mathematically in Equation S1. We assume that the high-charge-density ions drag a chain of water molecules into the oil phase containing water molecules, and the concentration of water molecules in the oil phase determines the probability (equivalent to the number of microstates) that the ion-water finger complex finds it. So, we obtain the formula S2, where Ω_w and Ω_o represent water concentrations in aqueous and organic phases, respectively. Obviously, the former is a constant value.

$$\Delta G_{\mathrm{tr},i}^{\circ,\mathrm{w}\to\mathrm{o}} = \Delta H_{\mathrm{tr},i}^{\circ,\mathrm{w}\to\mathrm{o}} - T\Delta S_{\mathrm{tr},i}^{\circ,\mathrm{w}\to\mathrm{o}} \quad \text{(S1)}$$

$$\Delta G_{\mathrm{tr},i}^{\circ,\mathrm{w}\to\mathrm{o}} = \Delta H_{\mathrm{tr},i}^{\circ,\mathrm{w}\to\mathrm{o}} - Tk_{\mathrm{B}}\ln\frac{\Omega_{\mathrm{o}}}{\Omega_{\mathrm{w}}} \quad \text{(S2)}$$

If the water contents of the two organic solvents are c_{w1} and c_{w2} respectively, and if $c_{w1} < c_{w2}$, then $\Omega_{o1} < \Omega_{o2}$. And $\Omega_w \gg \Omega_{o2} > \Omega_{o1}$, we can arrive at,

$$\Delta H_{\mathrm{tr},i}^{\circ,\mathrm{w}\to\mathrm{o1}} - Tk_{\mathrm{B}}\ln\frac{\Omega_{\mathrm{o1}}}{\Omega_{\mathrm{w}}} > \Delta H_{\mathrm{tr},i}^{\circ,\mathrm{w}\to\mathrm{o2}} - Tk_{\mathrm{B}}\ln\frac{\Omega_{\mathrm{o2}}}{\Omega_{\mathrm{w}}} \quad \text{(S3)}$$

$$\Delta G_{\mathrm{tr},i}^{\circ,\mathrm{w}\to\mathrm{o1}} > \Delta G_{\mathrm{tr},i}^{\circ,\mathrm{w}\to\mathrm{o2}} \quad \text{(S4)}$$

Substituting into equation 1 in the main text, we get

$\Delta_{\mathrm{o1}}^{\mathrm{w}}\varphi_i^{\circ} > \Delta_{\mathrm{o2}}^{\mathrm{w}}\varphi_i^{\circ}$ for cations (e.g., A^+) and $\Delta_{\mathrm{o1}}^{\mathrm{w}}\varphi_i^{\circ} < \Delta_{\mathrm{o2}}^{\mathrm{w}}\varphi_i^{\circ}$ for anions (e.g., B^-). Finally, $\mathrm{PPW}_{\mathrm{o1}}^{\mathrm{w}}$ (A^+ B^-) is wider than $\mathrm{PPW}_{\mathrm{o2}}^{\mathrm{w}}$ (A^+ B^-). Now, combining Equations S3 and S4, we can get

$$\mathrm{PPW}(\mathrm{A^+B^-}) \propto -\ln\Omega_{\mathrm{o}} \propto -\ln c_{\mathrm{w}} \quad \text{(S5)}$$

Table S1

Equilibrium molar concentration of water in organic solvents $c_w(mol/L) = {}^{c_w(ppm)\rho}/_{1.8 \times 10^4}$, molar concentration of solvent $c_s = {}^{1000\rho}/_{M}$, the Stear-Eyring diameter of the solvent molecule $d_s = ({}^{M}/_{10^6 N_A \rho})^{1/3}$ (N_A is the Avogadro number).[3] Notes: ρ, M, and p are the density, the molar mass, and the dipole moment of the solvents, respectively.

Solvents	M (g/mol)	ρ (g/mL)	ε_0	ε_1	p (D)	c_w (mol/L)	c_s (mol/L)	d_s (nm)
DCE	98.96	1.246	10.2	2.09	1.9	0.11[a]	2.59	0.510
DCM	84.933	1.325	9.1	2.03	1.34	0.111[b]	15.6	0.474
1,4-DCBu	127.01	1.1314	9.56	2.11	2.22	0.076[c]	8.93	0.570
1,6-DCH	155.06	1.065	8.83	2.12	2.47	0.057[c]	6.87	0.624
Nitroethane	75.067	1.009	30.3	1.93	3.23	0.841[b]	13.44	0.498
1-Nitropropane	89.09	1.0	24.7	1.96	3.66	0.333[b]	11.22	0.529
2-Nitropropane	89.09	0.992	26.74	1.94	3.73	0.276[b]	11.13	0.530
TCM	119.38	1.48	4.81	2.10	1.04	0.063[b]	12.40	0.512
CCl_4	153.823	1.594	2.24	2.13	0	0.0077[b]	10.36	0.543
DCB	147.002	1.306	10.1	2.40	2.14	0.022[d]	8.88	0.572
TFT	146.11	1.19	9.47	2.00	2.86	0.025[d]	8.14	0.588
NB	123.109	1.205	34.8	2.41	4.22	0.2[a]	9.79	0.554
CB	112.557	1.1075	5.6895	2.32	1.69	0.0196[b]	9.84	0.553
Aniline	93.127	1.022	7.06	2.52	1.13	3.5202[e]	10.97	0.533
Toluene	92.14	0.872	2.379	2.25	0.375	0.023[b]	9.46	0.560
NPOE	251.33	1.041	24.2	2.27	4.33	0.046[a]	4.14	0.738
5-nonanone	142.239	0.816	10.6	2.00	3.23	0.221[f]	5.74	0.661
MIBK	100.16	0.8	18.4	1.94	3.32	0.939[g]	7.99	0.592
n-ocT	130.2	0.83	7.36	10.3	1.68	2.26[h]	6.37	0.639
Ick	114.185	0.82	11.9	1.97	2.59	0.569[f]	7.18	0.614
dpk	114.185	0.82	11.9	1.97	3.26	0.375[f]	7.18	0.614

Solvents	M (g/mol)	ρ (g/mL)	ε_0	ε_1	p (D)	c_w (mol/L)	c_s (mol/L)	d_s (nm)
2-nonanone	142.24	0.886	9.14	2.00	3.16	0.353[f]	6.23	0.644
MHK	128.22	0.818	9.51	1.99	2.7	0.45[f]	6.38	0.638

Abbreviation and full name in the 1st column: DCE: 1,2-dichloroethane; DCM: dichloromethane; 1,4-DCBu: 1,4-dichlorobutane; 1,6-DCH: 1,6-dichlorohexane; TCM: trichloromethane; CCl_4: carbon tetrachloride; DCB: 1,2-dichlorobenzene; TFT: α,α,α-trifluorotoluene; NB: nitrobenzene; CB: chlorobenzene; NPOE: o-nitrophenyl octyl ether; MIBK: methyl isobutyl ketone; n-ocT: n-octanol; Ick: 2-heptanone; Dpk: 4-heptanone; MHK: 2-octanone.

[a]reported in ref.[4]

[b]recalculated from the data in ref.[5]

[c]recalculated from the data in ref.[6]

[d]reported in ref.[7]

[e]recalculated from the data in ref.[8]

[f]recalculated from the data in ref.[9]

[g]recalculated from the data in ref.[10]

[h]recalculated from the data in ref.[11]

Table S2

Some parameters of Li^+ and Cl^- ions.

Ions	a (nm)	hydration number	ΔG_n^0(exp) (kJ mol $^{-1}$)	ΔG_h^0(th)[c] (kJ mol $^{-1}$)
Li^+	0.078[a]	4	34.3	–545
Cl^-	0.181[b]	6	25.27	–286.3

[a]ref.[12]

[b]ref.[13]

[c]ref.[14]

Table S3

Comparison between the theoretical standard Gibbs free energy $\Delta G^{\circ,\mathrm{w}\to\mathrm{o}}_{\mathrm{tr,Li^+}}(\mathrm{th})$ of Li^+ (a = 0.078 nm) transferred from water (w) to organic solvents (o) and the experimental value $\Delta G^{\circ,\mathrm{w}\to\mathrm{o}}_{\mathrm{tr,Li^+}}(\mathrm{exp})$, where $\bar{\varepsilon_1} = (2n_w + \varepsilon_1 n_s)/n$.

solvent	c_w (mol/L)	$n_w(n_s)$	b (nm)	$\bar{\varepsilon_1}$	$\Delta G^{\circ,\mathrm{w}\to\mathrm{o}}_{\mathrm{tr,Li^+}}(\mathrm{th})$ (kJ/mol)	$\Delta G^{\circ,\mathrm{w}\to\mathrm{o}}_{\mathrm{tr,Li^+}}(\mathrm{exp})$ (kJ/mol)
DCB	0.022	0.8(3.2)	0.428	2.32(2.0)	69	83[b]
TFT	0.025	0.9(3.1)	0.436	2.0	71	78[c]
NB	0.2	2.7(1.3)	0.337	2.13	16	36[d]
CB	0.0196	0.7(3.3)	0.418	2.26	38	/
Aniline	3.5202	3.9(0.1)	0.257	2.0	26[a]	/
Toluene	0.023	0.8(3.2)	0.419	2.2	88	78[e]
NPOE	0.046	2.1(1.9)	0.470	2.13	44	47[f]

[a]equation (9) in the main text is used to calculate the electrostatic term of Gibbs solvation energy of an ion, ΔG°_{el}

[b]ref.[3]

[c]ref.[15]

[d]ref.[16]

[e]ref.[7]

[f]ref.[17]

Table S4

Comparison of the theoretical standard Gibbs free energy $\Delta G^{\circ,\mathrm{w}\to\mathrm{o}}_{\mathrm{tr,Cl^-}}(\mathrm{th})$ of Cl^- (a = 0.181 nm) transferred from water (w) to organic solvents (o) and the experimental value $\Delta G^{\circ,\mathrm{w}\to\mathrm{o}}_{\mathrm{tr,Cl^-}}(\mathrm{exp})$, where $\bar{\varepsilon_1} = (2n_w + \varepsilon_1 n_s)/n$. Note that the last two columns (from the left) are theoretical ($\Delta\phi(\mathrm{th})$) and experimental ($\Delta\phi(\mathrm{exp})$) values of the polarizable potential window width of LiCl at the w/o interfaces, in which the potential scale of Li^+ transfer at w/o is converted with the data of standard Gibbs free energy of Li^+ transfer as listed in Table S3. The experimental PPWs in the present work are shown in parentheses in the last column.

solvent	c_w (mol/L)	$n_w(n_s)$	b (nm)	$\bar{\varepsilon_1}$	$\Delta G^{\circ,\mathrm{w}\to\mathrm{o}}_{\mathrm{tr,Cl^-}}(\mathrm{th})$ (kJ/mol)	$\Delta G^{\circ,\mathrm{w}\to\mathrm{o}}_{\mathrm{tr,Cl^-}}(\mathrm{exp})$ (kJ/mol)	$\Delta\phi(\mathrm{LiCl,th})$ (V)	$\Delta\phi(\mathrm{LiCl,exp})$ (V)
DCB	0.022	1.2(4.8)	0.497	2.32(2.0)	64	32[b]	1.38	1.19(1.104)
TFT	0.025	1.4(4.6)	0.524	2.0	66	65.6[c]	1.42	1.49(1.14)
NB	0.2	4(2)	0.399	2.13	31	31[d]	0.49	0.69(0.566)
CB	0.0196	1(5)	0.487	2.27	59	/	1.0	/
Aniline	3.5202	5.2(0.8)	0.315	2.0	32[a]	/	0.6	/
Toluene	0.023	1.2(4.8)	0.487	2.2	97	4[e]	1.9	1.05
NPOE	0.046	3.2(2.8)	0.541	2.13	53	46.6[f]	1.0	0.97(0.504)

[a]equation (9) in the main text is used to calculate the electrostatic term of Gibbs solvation energy of an ion, ΔG°_{el}

[b]ref.[18]

[c]ref.[15]

[d]ref.[19]

[e]ref.[7]

[f]ref.[20]

Table S5

The comparison between the theoretical standard Gibbs free energy, $\Delta G^{\circ,\mathrm{w}\to\mathrm{o}}_{\mathrm{tr,Li^+}}(\mathrm{th})$, for the transfer of Li^+ (a = 0.078 nm) from water (w) to various organic solvents (o) and the experimental value, $\Delta G^{\circ,\mathrm{w}\to\mathrm{o}}_{\mathrm{tr,Li^+}}(\mathrm{exp})$, where $\overline{\varepsilon_1} = (2n_w + \varepsilon_1 n_s)/n$.

solvent	c_w (mol/L)	$n_w(n_s)$	b (nm)	$\overline{\varepsilon_1}$	$\Delta G^{\circ,\mathrm{w}\to\mathrm{o}}_{\mathrm{tr,Li^+}}(\mathrm{th})$ (kJ/mol)		$\Delta G^{\circ,\mathrm{w}\to\mathrm{o}}_{\mathrm{tr,Li^+}}(\mathrm{exp})$ (kJ/mol)
					Partially hydrated	Fully hydrated	
5-nonanone	0.221	3.2(0.8)	0.530	2.0	81	34	10.56[a]
MIBK	0.939	3.7(0.3)	0.280	2.0	23	22	20.26[b]
n-ocT	2.26	3.9(0.1)	0.263	2.0	38	23	11[c]
Ick	0.569	3.6(0.4)	0.294	2.0	36	31	/
dpk	0.375	3.4(0.6)	0.312	2.0	41	38	/
2-nonanone	0.353	3.4(0.6)	0.321	2.0	49	44	/
MHK	0.45	3.5(0.5)	0.310	2.0	45	40	/

[a]ref.[18]

[b]ref.[21]

[c]ref.[20]

Table S6

The comparison between the theoretical standard Gibbs free energy, $\Delta G^{\circ,w\rightarrow o}_{tr,Cl^-}(\mathrm{th})$, for the transfer of Cl^- (a = 0.181 nm) from water (w) to various organic solvents (o) and the experimental value, $\Delta G^{\circ,w\rightarrow o}_{tr,Cl^-}(\mathrm{exp})$, where $\bar{\varepsilon_1} = (2n_w + \varepsilon_1 n_s)/n$. Note that the last two columns (from the left) are theoretical ($\Delta\phi(\mathrm{th})$, these values are based on fully hydrated ions) and experimental ($\Delta\phi(\mathrm{exp})$) values of the polarizable potential window width of LiCl at the w/o interfaces, in which the potential scale of Li^+ transfer at w/o is converted with the data of standard Gibbs free energy of Li^+ transfer as listed in Table S5. The experimental PPWs in the present work are shown in parentheses in the last column.

solvent	c_w (mol/L)	$n_w(n_s)$	b (nm)	$\bar{\varepsilon_1}$	$\Delta G^{\circ,w\rightarrow o}_{tr,Cl^-}(\mathrm{th})$ (kJ/mol)		$\Delta G^{\circ,w\rightarrow o}_{tr,Cl^-}(\mathrm{exp})$ (kJ/mol)	$\Delta\phi(\mathrm{LiCl,th})$ (V)	$\Delta\phi(\mathrm{LiCl,exp})$ (V)
					Partially hydrated	Fully hydrated			
5-nonanone	0.221	4.8(1.2)	0.406	2.0	51	48	32.81[a]	0.85	0.45(0.679)
MIBK	0.939	5.5(0.5)	0.34	2.0	29	28	50.17[b]	0.52	0.73
n-ocT	2.26	5.8(0.2)	0.324	2.0	34	29	35[c]	0.54	0.48
Ick	0.569	5.0(1.0)	0.358	2.0	39	37	/	0.70	/
dpk	0.375	5.1(0.9)	0.377	2.0	44	40	/	0.81	/
2-nonanone	0.353	5.1(0.9)	0.380	2.0	49	44	/	0.91	/
MHK	0.45	5.3(0.7)	0.364	2.0	45	41	/	0.84	/

[a]ref.[18]

[b]ref.[21]

[c]ref.[22]

References

1. Liu, J.; Zheng, X.; Hua, Y.; Deng, J.; He, P.; Yu, Z.; Zhang, X.; Shi, X.; Shao, Y., Electrochemical Study of Ion Transfers Processes at the Interfaces between Water and Trifluorotoluene and Its Derivatives. *ChemElectroChem* **2022,** *9* (13), e202200389.

2. Williams, D. B. G.; Lawton, M., Drying of Organic Solvents: Quantitative Evaluation of the Efficiency of Several Desiccants. *J. Org. Chem.* **2010,** *75* (24), 8351-8354.

3. Trojánek, A.; Mareček, V.; Fiedler, J.; Samec, Z., Origin of the correlation between the standard Gibbs energy of ion transfer and the solubility of water in organic solvents. *Electrochim. Acta* **2023,** *465*, 142966.

4. Samec, Z.; Langmaier, J.; Trojánek, A., Polarization phenomena at the water | o-nitrophenyl octyl ether interface. Part 1. Evaluation of the standard Gibbs energies of ion transfer from the solubility and voltammetric measurements. *J. Electroanal. Chem.* **1996,** *409* (1), 1-7.

5. IUPAC-NIST Solubility Database, Version 1.1. NIST Standard Reference Database 106. http://dx.doi.org/10.18434/T4QC79.

6. Sabela, A.; Mareček, V.; Samec, Z.; Fuoco, R., Standard Gibbs energies of transfer of univalent ions from water to 1,2-dichloroethane. *Electrochim. Acta* **1992,** *37* (2), 231-235.

7. Zhang, J.; Huang, L.; Fang, T.; Du, F.; Xiang, Z.; Zhang, J.; Chen, R.; Peljo, P.; Ouyang, G.; Deng, H., Discrete Events of Ionosomes at the Water/Toluene Micro-Interface. *ChemElectroChem* **2022,** *9* (22), e202200624.

8. Góral, M.; Shaw, D. G.; Mączyński, A.; Wiśniewska-Gocłowska, B.; Oracz, P., IUPAC-NIST Solubility Data Series. 96. Amines with Water Part 3. Non-Aliphatic Amines. *J. Phys. Chem. Ref. Data* **2012,** *41* (4), 043108.

9. Góral, M.; Wiśniewska-Gocłowska, B., IUPAC-NIST Solubility Data Series. 86. Ethers and Ketones with Water. Part 6. C7–C12 Ketones with Water. *J. Phys. Chem. Ref. Data* **2008,** *37* (3), 1611-1653.

10. Góral, M.; Wiśniewska-Gocłowska, B., IUPAC-NIST Solubility Data Series. 86. Ethers and Ketones with Water. Part 5. C6 Ketones with Water. *J. Phys. Chem. Ref. Data* **2008,** *37* (3), 1575-1609.

11. Lang, B. E., Solubility of Water in Octan-1-ol from (275 to 369) K. *J. Chem. Eng. Data* **2012,** *57* (8), 2221-2226.

12. Goldschmidt, V. M., Krystallbau und chemische Zusammensetzung. *Berichte der deutschen chemischen Gesellschaft (A and B Series)* **1927,** *60* (5), 1263-1296.

13. Abraham, M. H.; Liszi, J., Calculations on ionic solvation. Part 1.—Free energies of solvation of gaseous univalent ions using a one-layer continuum model. *Journal of the Chemical Society, Faraday Transactions 1: Physical Chemistry in Condensed Phases* **1978,** *74* (0), 1604-1614.

14. Abraham, M. H.; Liszi, J., Calculations on ionic solvation—V The calculation of partition coefficients of ions. *Journal of Inorganic and Nuclear Chemistry* **1981,** *43* (1), 143-151.

15. Trojánek, A.; Mareček, V.; Langmaier, J.; Samec, Z., Effect of water solubility in organic solvents on the standard Gibbs energy of ion transfer across a water/organic solvent interface. *Electrochim. Acta* **2023,** *449*, 142222.

16. Samec, Z.; Mareček, V.; Colombini, M. P., Standard Gibbs energies of transfer of alkali metal cations from water to 1,2-dichloroethane: A critique. *J. Electroanal. Chem.* **1988,** *257* (1), 147-154.

17. Ulmeanu, S. M.; Jensen, H.; Samec, Z.; Bouchard, G.; Carrupt, P.-A.; Girault, H. H., Cyclic voltammetry of highly hydrophilic ions at a supported liquid membrane. *J. Electroanal. Chem.* **2002,** *530* (1), 10-15.

18. Deng, H.; Peljo, P.; Huang, X.; Smirnov, E.; Sarkar, S.; Maye, S.; Girault, H. H.; Mandler, D., Ionosomes: Observation of Ionic Bilayer Water Clusters. *J. Am. Chem. Soc.* **2021,** *143* (20), 7671-7680.
19. Scholz, F.; Komorsky-Lovrić, Š.; Lovrić, M., A new access to Gibbs energies of transfer of ions across liquid|liquid interfaces and a new method to study electrochemical processes at well-defined three-phase junctions. *Electrochem. Commun.* **2000,** *2* (2), 112-118.
20. Quentel, F.; Mirčeski, V.; Elleouet, C.; L'Her, M., Studying the Thermodynamics and Kinetics of Ion Transfers Across Water-2-nitrophenyloctyl Ether Interface by Means of Organic-solution-modified Electrodes. *J. Phys. Chem. C* **2008,** *112* (39), 15553-15561.
21. Koczorowski, Z.; Geblewicz, G.; Paleska, I., Electrochemical study of the water—isobutylmethyl ketone interface. *J. Electroanal. Chem.* **1984,** *172* (1), 327-337.
22. Gulaboski, R.; Mirčeski, V.; Scholz, F., An electrochemical method for determination of the standard Gibbs energy of anion transfer between water and n-octanol. *Electrochem. Commun.* **2002,** *4* (4), 277-283.
